# Quantitative analysis of the time-course of viral DNA forms during the HIV-1 life cycle

**DOI:** 10.1186/1742-4690-10-87

**Published:** 2013-08-13

**Authors:** Soundasse Munir, Sylvain Thierry, Frédéric Subra, Eric Deprez, Olivier Delelis

**Affiliations:** 1 LBPA, ENS Cachan, CNRS, Cachan, France

**Keywords:** HIV-1, 3′-processing, 1-LTR circles, Integrase, Strand transfer inhibitors

## Abstract

**Background:**

HIV-1 DNA is found both integrated in the host chromosome and unintegrated in various forms: linear (DNA_L_) or circular (1-LTRc, 2-LTRc or products of auto-integration). Here, based on pre-established strategies, we extended and characterized in terms of sensitivity two methodologies for quantifying 1-LTRc and DNA_L_, respectively, the latter being able to discriminate between unprocessed or 3′-processed DNA.

**Results:**

Quantifying different types of viral DNA genome individually provides new information about the dynamics of all viral DNA forms and their interplay. For DNA_L_, we found that the 3′-processing reaction was efficient during the early stage of the replication cycle. Moreover, strand-transfer inhibitors (Dolutegravir, Elvitegravir, Raltegravir) affected 3′-processing differently. The comparisons of 2-LTRc accumulation mediated by either strand-transfer inhibitors or catalytic mutation of integrase indicate that 3′-processing efficiency did not influence the total 2-LTRc accumulation although the nature of the LTR-LTR junction was qualitatively affected. Finally, a significant proportion of 1-LTRc was generated concomitantly with reverse transcription, although most of the 1-LTRc were produced in the nucleus.

**Conclusions:**

We describe the fate of viral DNA forms during HIV-1 infection. Our study reveals the interplay between various forms of the viral DNA genome, the distribution of which can be affected by mutations and by inhibitors of HIV-1 viral proteins. In the latter case, the quantification of 3′-processed DNA in infected cells can be informative about the mechanisms of future integrase inhibitors directly in the cell context.

## Background

After HIV-1 particles enter their target cells, reverse transcriptase converts HIV-1 viral RNA into a double-stranded linear DNA (DNA_L_). The resulting DNA_L_ moves into the nucleus as a component of the pre-integration complex (PIC) and then integrates into the host cell genome [1]. Integration of DNA_L_ is essential for a productive infection [2,3]. This process requires the integrase activity for the 3′-processing reaction at both LTR (Long Terminal Repeat) extremities of the blunt (or unprocessed) DNA, named uDNA_L_, leading to 3′-processed linear DNA (pDNA_L_) [4]. This reaction involves the removal of a dinucleotide after the canonical 5′-CA found in all retroviruses. The integrase-mediated integration of pDNA_L_ into the host cell genome can be efficiently inhibited by integrase strand-transfer inhibitors (INSTIs) [5], including Raltegravir (RAL), Dolutegravir (DTG) and Elvitegravir (EVG); this inhibition is similar to that associated with inactivation of the catalytic triad of integrase (for example due to D116N/A mutation [6,7]). It is important to note that the INSTI compounds, unlike catalytic mutants, are not supposed to influence the 3′-processing reaction and inhibit the strand transfer reaction of the integration process only [8]. It is intriguing to note that the context of catalytic mutant or INSTI treatment lead to similar 2-LTRc accumulation despite differentially affecting the 3′-processing step [9,10]. One possible explanation is that although INSTI are specific strand transfer inhibitors *in vitro*, they may affect the 3′-processing reaction in the cell context. Alternatively, the formation of 2-LTRc could be compatible with both types of viral DNA ends, processed or unprocessed leading to both 2-LTRc encompassing a perfect palindromic junction (resulting from ligation of blunt extremities) and others with an imperfect palindromic junction (most likely originating from auto-integration [11]). However, this issue remains unresolved because quantitative and sensitive data about the fate of linear HIV-1 DNA, more particularly for separately quantifying the two principal forms of linear DNA (pDNA_L_ and uDNA_L_), are not well-established.

In addition to DNA_L_ (=pDNA_L_ + uDNA_L_), unintegrated forms of HIV-1 DNA include DNA circles which harbor one or two LTR (1-LTRc and 2-LTRc, respectively); 1-LTRc is formed by circularization of DNA_L_ by homologous recombination and 2-LTRc by the Non-Homologous End Joining (NHEJ) pathway [12-14]. Other circular forms, resulting from auto-integration, could also be detected using PCR assays [15]. For instance, in the context of 2-LTRc, auto-integration events lead to 2-LTRc harboring imperfect palindromic junction in contrast to 2-LTRc originating from NHEJ [11]. Although 2-LTRc are considered to be dead-end molecules, 1-LTRc may sustain viral gene expression [13,16].

To date, few quantitative data about the intracellular localization of HIV-1 DNA species are available. Moreover, the relative abundance of the different viral DNA forms is dynamic and is dependent of viral conditions of infection. The intracellular localization of viral genomes has been determined by Southern blotting experiments [17]. However, this approach suffers from a lack of detection sensitivity and the distribution of viral forms can be assessed only qualitatively. Although real-time PCR-based protocols have been developed for accurately quantifying the integrated, 2-LTRc and total viral DNA forms [18,19], no method for accurate quantification of 1-LTRc is available [20]. Concerning DNA_L_, based on previous established strategy [21], we explored the optimal conditions for reliable quantification and further characterized the quantification of pDNA_L_ and uDNA_L_, and compared the sensitivity of this approach to that of Southern blotting.

We report original information related to the dynamics of all viral DNA genomes, and the efficiency and localization of the 3′-processing reaction. We also described the action of the anti-integrase compounds. Mainly, we found that 3′-processing is an efficient process (80% of linear DNA is processed) occurring in cytoplasm at early stage of the replication cycle, concomitant with or soon after reverse transcription. Furthermore, we show that INSTIs at sub-micromolar concentrations that fully prevent integration do not inhibit the 3′-processing reaction in the context of viral infection. Regarding circular forms, we observed that 1-LTRc are mainly formed in the nucleus but, unlike 2-LTRc which are exclusively generated in the nucleus, a small but significant proportion (10%) of 1-LTRc are formed in the cytoplasm.

## Results

**Figure 1:**
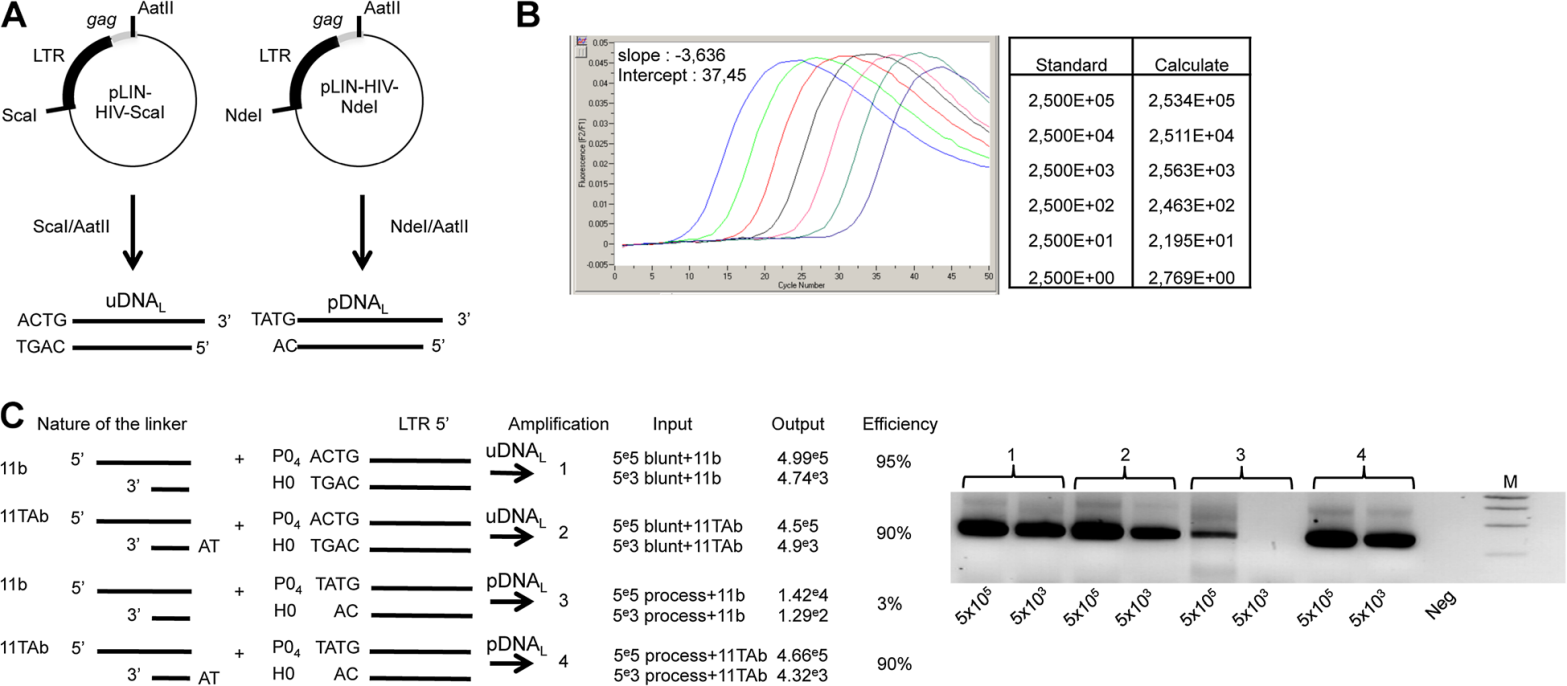
**Quantification of linear viral DNA. (A)** Plasmids pLIN-HIV-ScaI and pLIN-HIV-NdeI are depicted. These plasmids were used as controls for the reaction specificity. DNAs mimicking uDNA_L_ and pDNA_L_ were obtained by ScaI/AatII and NdeI/AatII digestion of pLIN-HIV-ScaI and pLIN-HIV-NdeI, respectively. **(B)** Amplification of serial dilutions of the pLIN-HIV-ScaI plasmid. Parameters of the PCR (slope of the regression curve and intercept) using primers 25 t and MS2 are shown in the figure. **(C)** Amplifications resulting from the LM-PCR protocol, using the two linkers 11b or 11TAb in combination with DNA mimicking uDNA_L_ or pDNA_L_. After ligation, all possible combinations were subjected to the LM-PCR protocol. The input was defined as the initial amount, determined by quantitative PCR. The output is the value obtained by quantification with the LM-PCR protocol. Efficiency is defined as the output:input ratio. The standard curves used for pDNA_L_ and uDNA_L_ quantifications in infected cells were obtained using 11TAb and 11b linkers, respectively (using serial dilutions of fragments obtained by NdeI/AatII and ScaI/AatII digestions of pLIN-HIV-NdeI and pLIN-HIV-ScaI, respectively). Right panel: PCR products from the various amplifications were loaded on agarose gel; M: Molecular weight marker.

To extend the quantitative analysis to other HIV-1 DNA forms, we addressed two quantitative protocols based on real-time PCR for quantifying linear viral DNA and 1-LTRc. Regarding linear viral DNA, we used a linker-mediated PCR method based on a qualitative approach described by Pierson and colleagues [22] (see Methods). This method involves using the 11TAb linker to detect total HIV-1 linear DNA (i.e. the processed, pDNA_L_, and unprocessed, uDNA_L_) and the 11b linker to detect uDNA_L_ only (Figure 1). The quantification of both linear DNA forms was reliable as attested by the good performance in terms of efficiency and sensitivity (90% efficiency over a 7-log range and a sensitivity of 100 DNA copies per 10^6^ cells) (Figure 1). This method was more sensitive than Southern blotting, for which the limit of detection was 10^5^ copies per 10^6^ cells (Additional file 1: Figure S1A). Thus, this method is suitable for quantitative analysis of the 3′-processing reaction in the virological context. It is important to note that, following infection, the overhanging dinucleotide of the processed HIV-1 DNA is 5′-AC (for the LTR5′ or LTR3′) and not the 5′-TA found in pDNA_L_ (resulting from the NdeI/AatII digestion of pLIN-HIV-NdeI). Thus, in order to extend the quantification procedure for DNA_L_ in the virological context, the linker 11GTb (with a TG-5′ overhanging; Additional file 1: Table S1A) was used rather than 11TAb in further experiments described below (the linker used for uDNA_L_ quantification was still 11b). The efficiency of the method using the linker 11GTb was verified (Additional file 1: Figure S1B).

**Figure 2:**
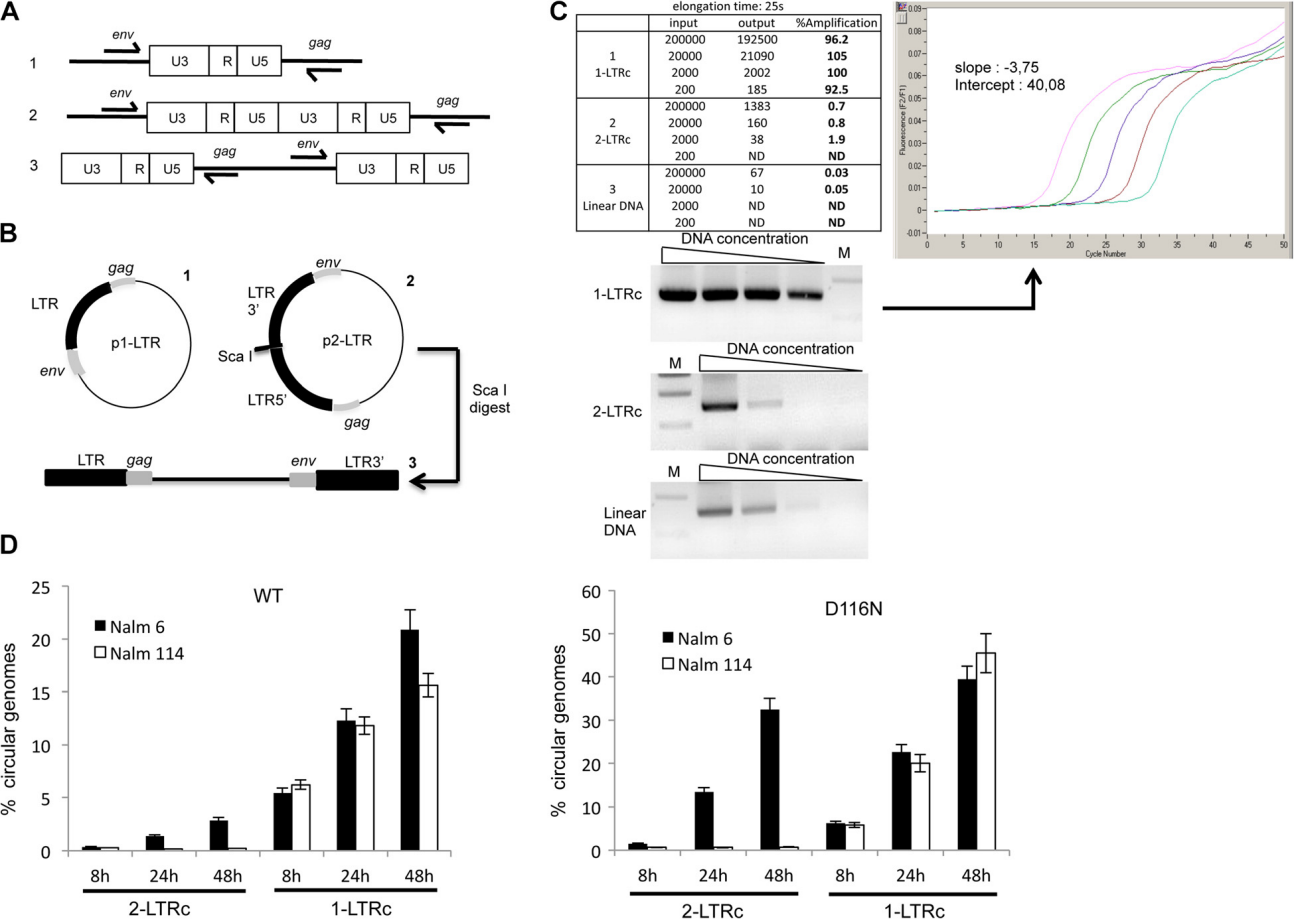
**Quantification of 1-LTRc. (A)** Possible amplifications from the various substrates found in infected cells with primers used for 1-LTRc quantification. 1: 1-LTRc; 2: 2-LTRc and 3: linear viral DNA. **(B)** Plasmids (1: p1-LTR and 2: p2-LTR) used as controls. Linear DNA (3) was obtained by ScaI digestion of p2-LTR. **(C)** Amplification of serial dilutions of p1-LTR using the protocol for 1-LTRc amplification. Amplification of known amounts of p1-LTR (1), p2-LTR (2) and linear DNA (3) using the protocol for 1-LTRc quantification. The results are reported in the table, for an elongation time of 25 s. Input: initial amount of target. Output: amount measured with the 1-LTRc protocol. %Amplification calculation is based on the output:input ratio. The plot shows the amplification results with known amounts of p1-LTR. The PCR products obtained with the various substrates were loaded onto an agarose gel (shown below the table). M: Molecular weight marker. **(D)** 1-LTRc quantification is not influenced by the presence of 2-LTRc. Nalm6 and Nalm114 cells were infected with VSV-G-pseudotyped NLENG1-ES-IRES WT (left panel) or NLENG1-ES-IRES D116N (right panel). At different times post-infection, the percentages of 2-LTRc and 1-LTRc were determined in Nalm6 (ligase-4+) (black columns) and Nalm114 (ligase-4-) (white columns) cell lines. Each value corresponds to an average of five to six independent experiments and confidence intervals analysis are shown for a p value <0.05.

Southern blotting analyses indicate that the amounts of 1-LTRc during infection are not negligible: 1-LTRc are more abundant than 2-LTRc and are present in similar amount compared to DNA_L_[23]. Thus, their accurate quantification is required to understand the relationships between the different viral DNA forms. Despite previous efforts to develop PCR-based methods for quantifying 1-LTRc [24,25], it has been reported that these methods are inaccurate [20]. Indeed, primers hybridizing in the *env* and *gag* genes could lead to amplification of the LTR-LTR region in 2-LTRc and amplification of DNA_L_ via LTR recombination [20] (see also Figure 2A). We therefore optimized an improved quantitative PCR protocol for 1-LTRc by addressing as a first criterion the detection of undesirable above-mentioned amplification products. We found that the elongation time was the crucial parameter to ensure specific amplification of 1-LTRc. Among the different tested conditions (modulation of the elongation time of the PCR), we found that 25 s was optimal (Additional file 1: Figure S2). Using p1-LTR for establishing a standard curve, we found that our protocol gave good amplification (92.5-100%) and provided sensitive detection (200 copies/10^6^ cells) of 1-LTRc (Figure 2). We then addressed the question of detection specificity and the influence of 2-LTRc content on the 1-LTRc quantification during infection by using Nalm-6 (ligase-4^+^) and Nalm-114 (ligase-4^-^) cells infected with HIV-1 Δenv viruses, either WT or D116N (a catalytic mutant of integrase [7]) [26]. It was previously described by Southern blot analysis that ligase-4 is involved in the formation of 2-LTRc only (not 1-LTRc) and that the D116N mutation leads to a substantial increase in the 2-LTRc content in the ligase-4^+^ context due to integration defect (to a much less extent the 1-LTRc content) [27]. Quantifications of total viral DNA as well as each circular viral DNA form (1-LTRc or 2-LTRc) were performed at different times post-infection (p.i.) (Figure 2D). Our results confirmed the strong inhibition (by a 40-fold factor) of 2-LTRc formation for both WT and D116N in Nalm-114 compared to Nalm-6 [12]. The amounts of 1-LTRc were similar in both cell lines infected by WT or D116N. This result confirms that the ligase 4 is not involved in the formation of 1-LTRc, consistent with the qualitative results reported by Li and colleagues [28]. Importantly, the amount of 1-LTRc was found to be similar regardless of the amount of 2-LTRc accumulated (compare in Figure 2D Nalm-6 and Nalm-114 infected by D116N). This confirms that our quantitative approach allows an accurate quantification of 1-LTRc in the cellular context without any bias due to the presence of 2-LTRc.

Based on (i) previously validated PCR-based protocols for quantification of total viral DNA, 2-LTRc and integrated viral DNA and (ii) the improvement described above for quantification of 1-LTRc, uDNA_L_ and pDNA_L_, we established the time course of these HIV-1 genomes during infection.

### Exhaustive time course study of viral DNA forms during infection

**Figure 3:**
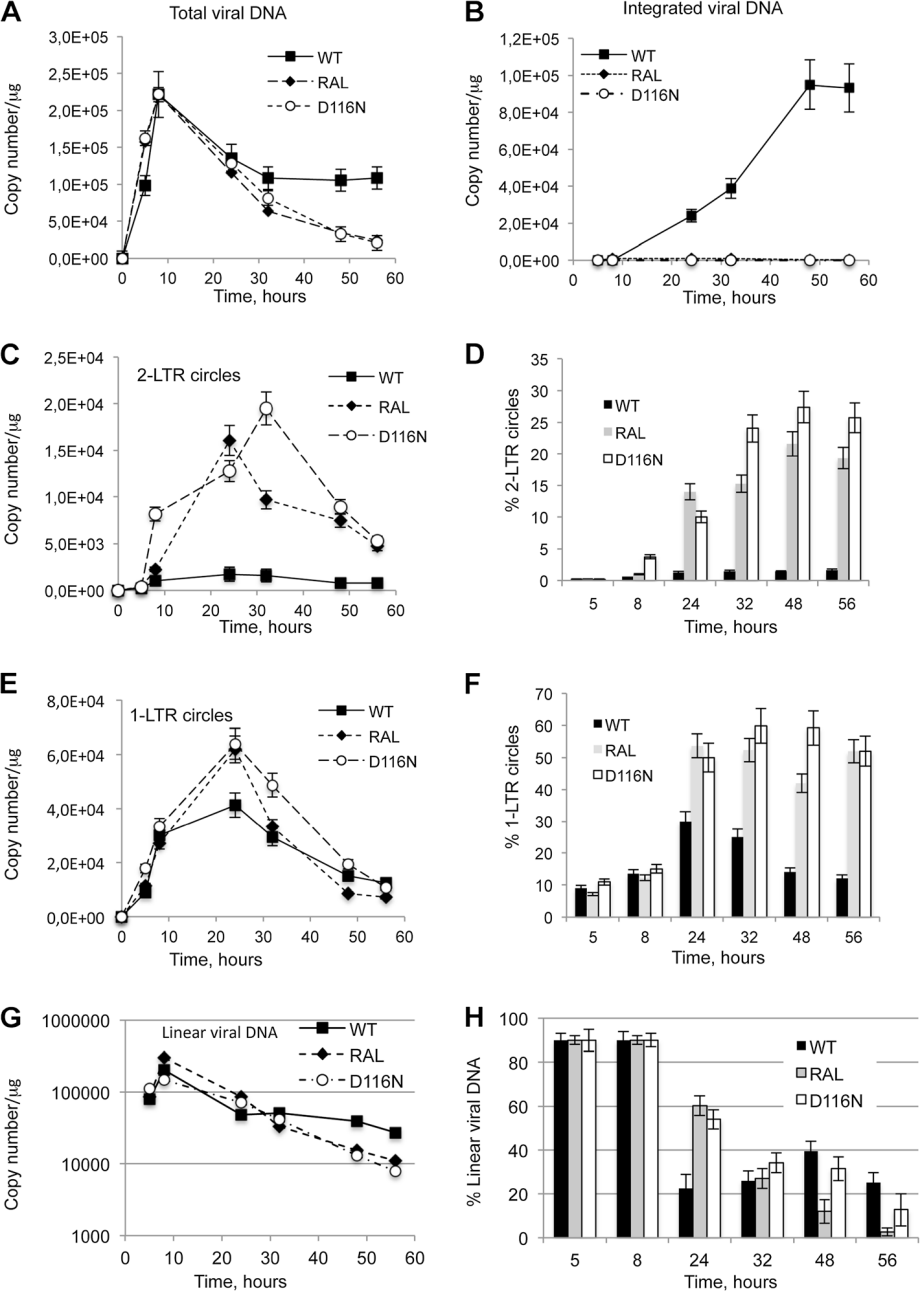
**Kinetics of total, circular, linear and integrated DNA forms during a single round of replication.** MT4 cells were infected with the VSV-G-pseudotyped NLENG1-ES-IRES WT (+/- RAL 500 nM) or NLENG1-ES-IRES D116N viruses. Each value corresponds to an average of five to six independent experiments and confidence intervals analysis are shown for a p value <0.05. **(A)** Total viral DNA. **(B)** Integrated viral DNA. **(C)** 2-LTRc. **(D)** Percentage of 2-LTRc (2-LTRc/Total viral DNAx100). **(E)** 1-LTRc. **(F)** Percentage of 1-LTRc (1-LTRc/Total viral DNA x 100). **(G)** Kinetics of total linear viral DNA (uDNA_L_ + pDNA_L_). WT: black squares; WT + 500 nM RAL: black diamonds; D116N: white circles. **(H)** Time course of linear viral DNA as a percentage of total viral DNA during infection. WT: black columns; WT + 500nM RAL: grey columns; D116N: white columns.

**Figure 4:**
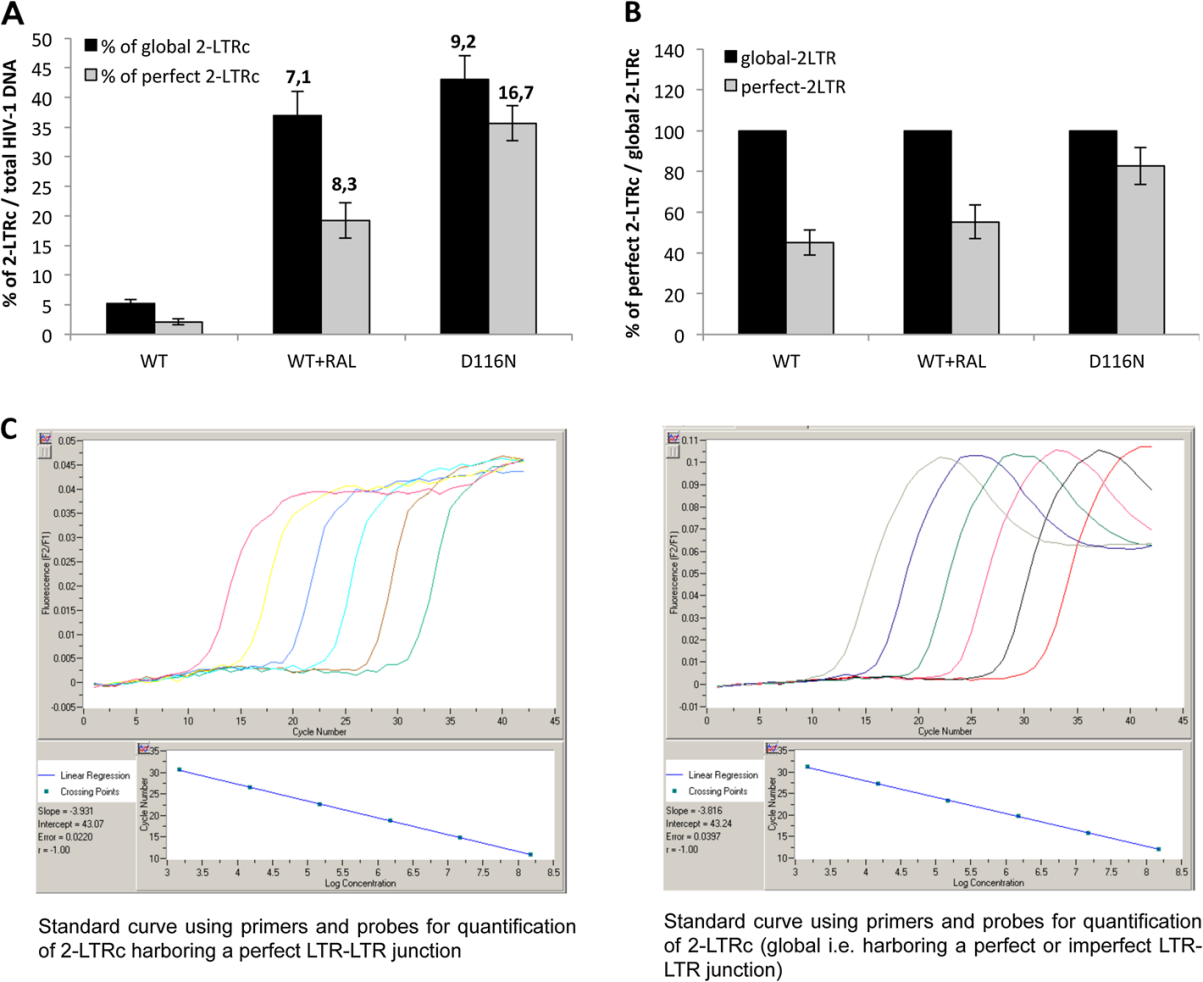
**Characterization of the nature of the LTR-LTR junction.** MT4 cells were infected with the VSV-G-pseudotyped NLENG1-ES-IRES WT (+/- RAL 500 nM) or NLENG1-ES-IRES D116N viruses. Each value corresponds to an average of five to six independent experiments and confidence intervals analysis are shown for a p value <0.05. **(A)** 2-LTRc as a percentage of total HIV-1 DNA 24 h post-infection (black column, global 2-LTRc and grey column, 2-LTRc harboring a perfect LTR-LTR junction). The fold increase in the amount of 2-LTRc (between integration defective conditions (WT + RAL and D116N) and the WT) is indicated at the top of the columns. **(B)** 2-LTRc harboring a perfect palindromic sequence as a percentage of total 2-LTRc in each condition of infection (WT, WT + RAL and D116N). **(C)** Standard curves with p2-LTR using primers and probes for quantification of 2-LTRc harboring a perfect LTR-LTR junction (left panel) and for quantification of total 2-LTRc (right panel).

We used MT4 cells infected with Δenv viruses (WT+/-RAL or D116N) as a model system to study the kinetics of viral DNA forms (integrated viral DNA, 1-LTRc, 2-LTRc and DNA_L_) during a single-round of viral replication [26]. Viral DNA was analyzed at different times p.i.. In the WT context, total HIV-1 DNA level peaked 8 h p.i. and then decreased steeply until 32 h p.i. (Figure 3A) to match the integrated HIV-1 DNA level (Figure 3B) as already reported [18]. Integration inhibition by RAL or due to the D116N mutation did not influence reverse transcription as total viral DNA synthesis was similar to the WT condition (Figures 3A and B). Under integration inhibition conditions, 2-LTRc accumulated to reach a maximum at 24 h-32 h p.i. whereas the amount of 2-LTRc in the WT context remained low (Figure 3C), as previously reported [8,29]. The study of circular forms behavior in the different conditions reveals two interesting observations. The first one is related to 1-LTRc: In the short time scale p.i. (until 5 h), significant 1-LTRc amount was detected while, in the same time, no 2-LTRc was detected. This result is intriguing because in a longer time scale p.i. (after 5 h), 1-LTRc synthesis followed 2-LTRc synthesis. The reason for such an apparent discrepancy will be further discussed (see next section). The second one concerns the similar accumulation of 2-LTRc in both conditions of IN inhibition: presence of RAL or D116N mutation. This observation is intriguing in first approximation because it is known that RAL and D116N differentially affect 3′-processing. Two hypotheses may explain such observations: (i) Either RAL behaves differentially in the virological context. (ii) The accumulated 2-LTRc are qualitatively different. Until now, 2-LTRc accumulation was studied using primers spanning the LTR-LTR junction, such that the nature of the LTR-LTR junction (representing global 2-LTRc) cannot be investigated. Recently, De Iaco et al. used oligonucleotides hybridizing at the palindromic junction, and discriminated 2-LTRc harboring a perfect palindromic sequence at the LTR-LTR junction (resulting from ligation of unprocessed extremities) from those harboring a deletion or insertion (imperfect palindrome, for instance formed by auto-integration) [11]. Using this setting, we confirm that the accumulation of global 2-LTRc was similar when integration was impaired by RAL treatment or by D116N mutation (Figure 4A). However, the accumulation of 2-LTRc harboring a perfect palindromic junction was similar, at about 40%, for WT and RAL conditions, but much higher, about 80%, in the case of D116N (Figure 4B). In conclusion, even though 3′-processing influences the nature of the palindromic junction, the global amount similarly increases when integration is inhibited, regardless of the 3′-processing status.

Yan et al. have shown that auto-integration occurs during reverse transcription leading to circular forms detected only during WT infection and not with RAL or the D116N/A mutant. Indeed, RAL or the D116N/A mutation abolish the auto-integration activity of integrase [15]. Our data suggest that the nature of the palindromic junction is influenced by 3′-processing but is not directly related to auto-integration. Indeed, the accumulation of 2-LTRc harboring an imperfect junction in the presence of RAL raises the question related to the relationship between these 2-LTRc forms and the auto-integration events since RAL is supposed to inhibit auto-integration. The mechanism behind the formation of these 2-LTRc forms remains to be elucidated.

The observed decreasing phase for circular forms corresponded to dilution due to cell division, in contrast to that observed for integrated forms (Figure 3B). Indeed, the absolute quantities of both 2-LTRc and 1-LTRc significantly decreased (Figures 3C and E) while their representativeness (normalized by total viral DNA) remained roughly constant (Figures 3D and Figure 3F, respectively). In the case of integrated forms, both their absolute quantity and their representativeness remained constant. A third behavior was observed with linear DNA: The maximal amount of DNA_L_ was obtained 8 h p.i. coinciding with the maximal amount of total viral DNA originating from reverse transcription (Figure 3G). After 8 h p.i., the amount of DNA_L_ decreased continuously. In contrast to that observed with viral DNA circular forms, the representativeness of DNA_L_ also decreased in all conditions of infections (WT+/- RAL and D116N). This indicates that DNA_L_ is less stable than 1-LTRc and 2-LTRc (Figure 3H) (for a detailed analysis of pDNA_L_ and uDNA_L_ stabilities, see section related to 3′-processing quantification). Intriguingly, along the decreasing phase characterizing the WT infection, we observed a reproducible rebound of DNA_L_ synthesis occurring between 32 and 48 h p.i., after a significant decreasing phase occurring between 8 and 24 h p.i.. Experiments are ongoing to explain this phenomenon.

Regarding the accumulation of circular DNA forms, it is important to note that impairing integration (RAL treatment or D116N infection) increased the accumulation of 1-LTRc and to a greater extent the accumulation of global 2-LTRc (compare Figures 3C and E). Accumulations of both 2-LTRc and 1-LTRc were also reflected in their relative representativeness (normalized by total viral DNA), but again, with a much more greater relative accumulation for 2-LTRc (10-fold) compared to 1-LTRc (2-fold) (Figures 3D and F). Indeed, in the RAL or D116N context, 2-LTRc reached 15-20% or 25-27% of total viral DNA, respectively, compared to 2% in the standard infection condition (Figure 3D), while 1-LTRc reached 50-60% of total viral DNA compared to 30% in the standard infection (Figure 3F). Moreover, our results indicate that the relative representativeness of 1-LTRc and 2-LTRc remained roughly constant from 32 h p.i. showing that the two circular viral DNA forms are relatively and equally stable.

### Two origins for 1-LTRc formation

**Figure 5:**
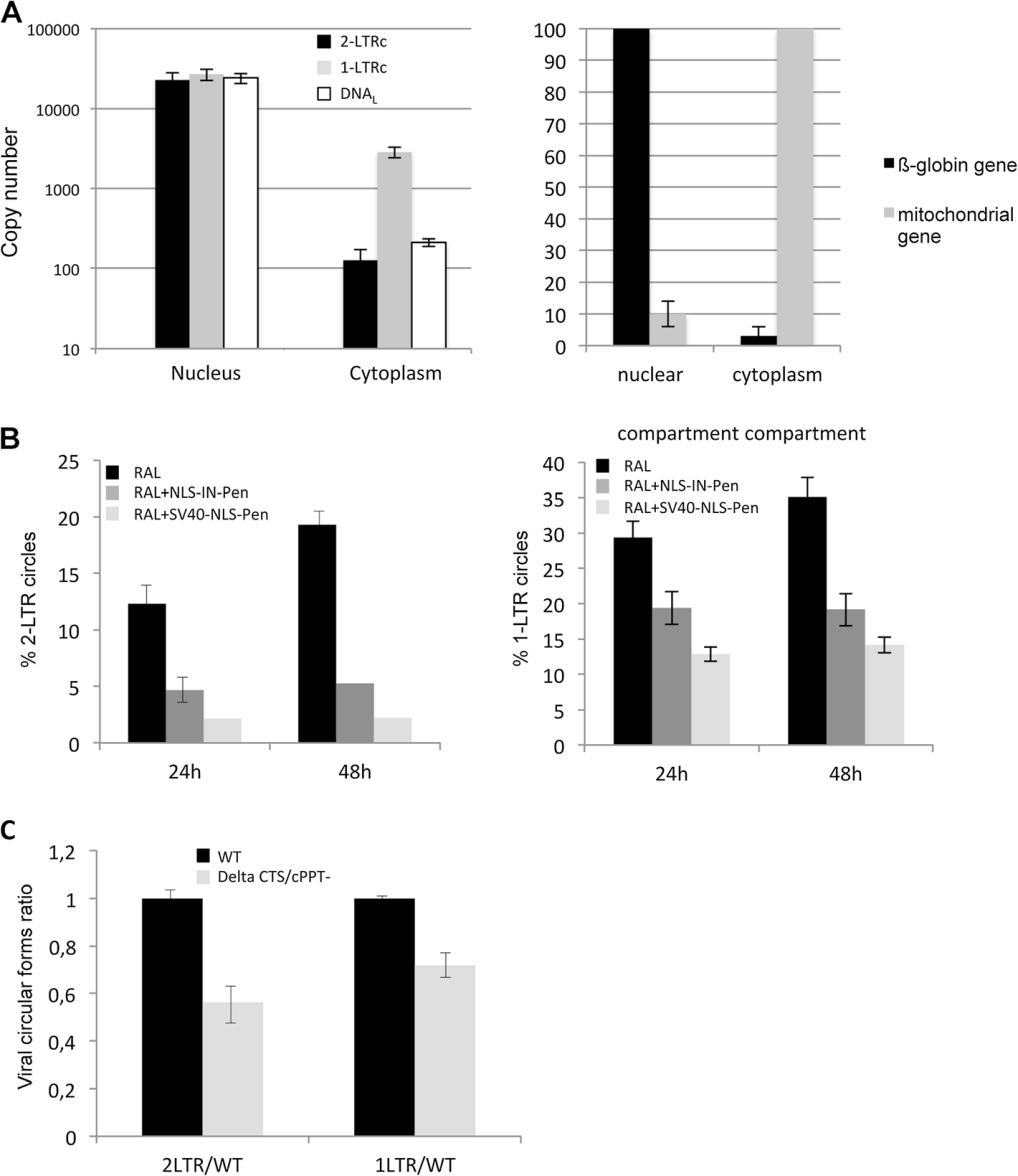
**Localization of 1-LTRc formation. (A)** Left panel, HeLa cells were infected cells with NLENG1-ES-IRES D116N and fractionated into a cytoplasmic and nuclear fraction 24 h post-infection. 2-LTRc (black columns), 1-LTRc (grey columns) and DNAL (white columns) were quantified in each fraction. Right panel, quantification of ß-globin gene and mitochondrial gene in the nuclear and cytoplasmic compartment. **(B)** HeLa cells were infected (WT + 500 nM RAL; black columns) in the presence of either 100 μM NLS-IN-Pen (dark grey columns) or 100 μM SV40-NLS-Pen (grey columns). Percentages of 2-LTRc (left panel) and 1-LTRc (right panel) at two times post-infection are shown. **(C)** HeLa cells were infected either with pNL4.3 virus (WT; black columns) or the CTS/cPPT mutant virus (grey columns). Ratios of 2-LTRc and 1-LTRc (the amount of 2-LTRc or 1-LTRc in the mutant condition divided by that for the WT at 24 h post-infection) are shown. Each value corresponds to an average of five to six independent experiments and confidence intervals analysis are shown for a p value <0.05.

As mentioned above, 1-LTRc were detected earlier than 2-LTRc in a context where their respective representativeness were comparable (D116N or WT + RAL) (Figure 3E). This prompted us to re-examine the question of subcellular location for 1-LTRc formation. To date, the origin of 1-LTRc formation remains unclear leading to apparent contradiction in the literature. For instance, Kilzer and colleagues reported that 1-LTRc formation requires homologous recombination between the two LTR of DNA_L_ which occurs in the nucleus [12]. By contrast, in an early study, Miller and colleagues proposed that 1-LTRc formation involves the reverse transcription step in the cytoplasm compartment [30]. We conducted cell fractionation experiments with MT4 cells 24 h p.i. infected with D116N (when amount of 2-LTRc is maximal; as 2-LTRc are formed exclusively in the nucleus, nuclear import has occurred at this time point [31]). ß-globin and mitochondrial genes quantifications were used as controls for cell fractionation validation (Figure 5A, right panel). Our results demonstrate that 2-LTRc were almost entirely detected in the nuclear fraction (>99.5%) (Figure 5A, left panel). The 1-LTRc amount was much more higher in the cytoplasmic fraction compared to 2-LTRc. Indeed, in conditions where the nuclear import is maximal, 1-LTRc formed in the cytoplasm represent 10% of total 1-LTRc (compared to <0.5% for 2-LTRc). This is consistent with the kinetics of 1-LTRc formation where 1-LTRc makes up 10% of total viral DNA as soon as 5 and 8 h p.i. (Figure 3E). These observations are compatible with the hypothesis of Miller and collaborators [30] that, at least, some of the 1-LTRc are produced during the reverse transcription step.

Peptides NLS-IN-Pen and SV40-NLS-Pen, previously described to inhibit the PIC nuclear import by inhibiting the Integrase-importinα interaction [32], were used to assess whether 1-LTRc are only formed during the reverse transcription step independently of PIC translocation. The amount of 1-LTRc was then measured and the nuclear import inhibition efficiency was monitored by quantification of 2-LTRc. HeLa cells, treated with either of these peptides, were infected with a pNL4.3 virus in the presence of RAL. In the absence of the peptides, 2-LTRc accumulated to 19.3% of total viral DNA 48 h p.i.. Peptide treatments led to an inhibition of 2-LTRc accumulation (5.22% and 2.24% for NLS-IN-Pen and SV40-NLS-Pen, respectively) confirming nuclear import inhibition (3.7 and 8.6 fold for NLS-IN-Pen and SV40-NLS-Pen, respectively) (Figure 5B, left panel). Interestingly, in these conditions, we observed a decrease in 1-LTRc formation but not to the same extent compared to 2-LTRc inhibition (1.8 and 2.5 fold for NLS-IN-Pen and SV40-NLS-Pen, respectively) (Figure 5B, right panel). It has been reported that inhibition of the PIC nuclear import can also be prevented more specifically by mutation in the FLAP and/or CTS region of the virus [23,33]. We infected HeLa cells with a defective mutant, affected in both the CTS and the PPT, and assayed 2-LTRc and 1-LTRc (relative to the WT condition) 24 h p.i. (Figure 5C). Disruption of the FLAP structure partially inhibited PIC nuclear import (and not fully as described in [23]), and was associated with the amount of 2-LTRc being reproducibly and significantly less than for the WT (about 2-fold). Such a decrease is compatible with previous findings indicating that mutants affected in the FLAP structure may replicate albeit slower than the WT [34-36]. In this context, we found a concomitant decrease of 1-LTRc but to a lesser extent (1.4-fold). Taken together, these data clearly suggest that the two mechanisms of 1-LTRc formation (and the two associated subcellular localizations) are not mutually exclusive: 1-LTRc can be formed in the cytoplasm during the reverse transcription step as previously suggested [30] but that most 1-LTRc (90%) is formed by homologous recombination after PIC translocation in the nucleus [12].

### Time course of the 3′-processing reaction in infected cells and study of the differential stability of 3′-processed and blunt linear viral DNAs

Regarding DNA_L_ quantification, the ability of our PCR-based protocol to further discriminate between both pDNA_L_ and uDNA_L_ forms, prompted us to study the kinetics of the 3′-processing reaction directly in infected cells. We used several conditions of infection: WT (permissive for both 3′-processing and integration processes), WT + RAL (permissive for 3′-processing reaction only, according to *in vitro* assays [37]) or D116N context (non permissive for both processes). At this stage, it is important to note that our protocol for quantifying total DNA_L_, pDNA_L_ and uDNA_L_ was validated using the LTR-5′ of the virus. We performed similar quantifications using specific primers and probes for the LTR-3′ (see Methods). The quantification of both pDNA_L_ and uDNA_L_ in infected MT4 cells led to similar values for 3′-processing activity at both LTR-3′ and LTR-5′ ends (Additional file 1: Figure S3), consistent with a previous report [38]. In the following study, pDNA_L_ and uDNA_L_ were then quantified on the LTR-5′.

**Figure 6:**
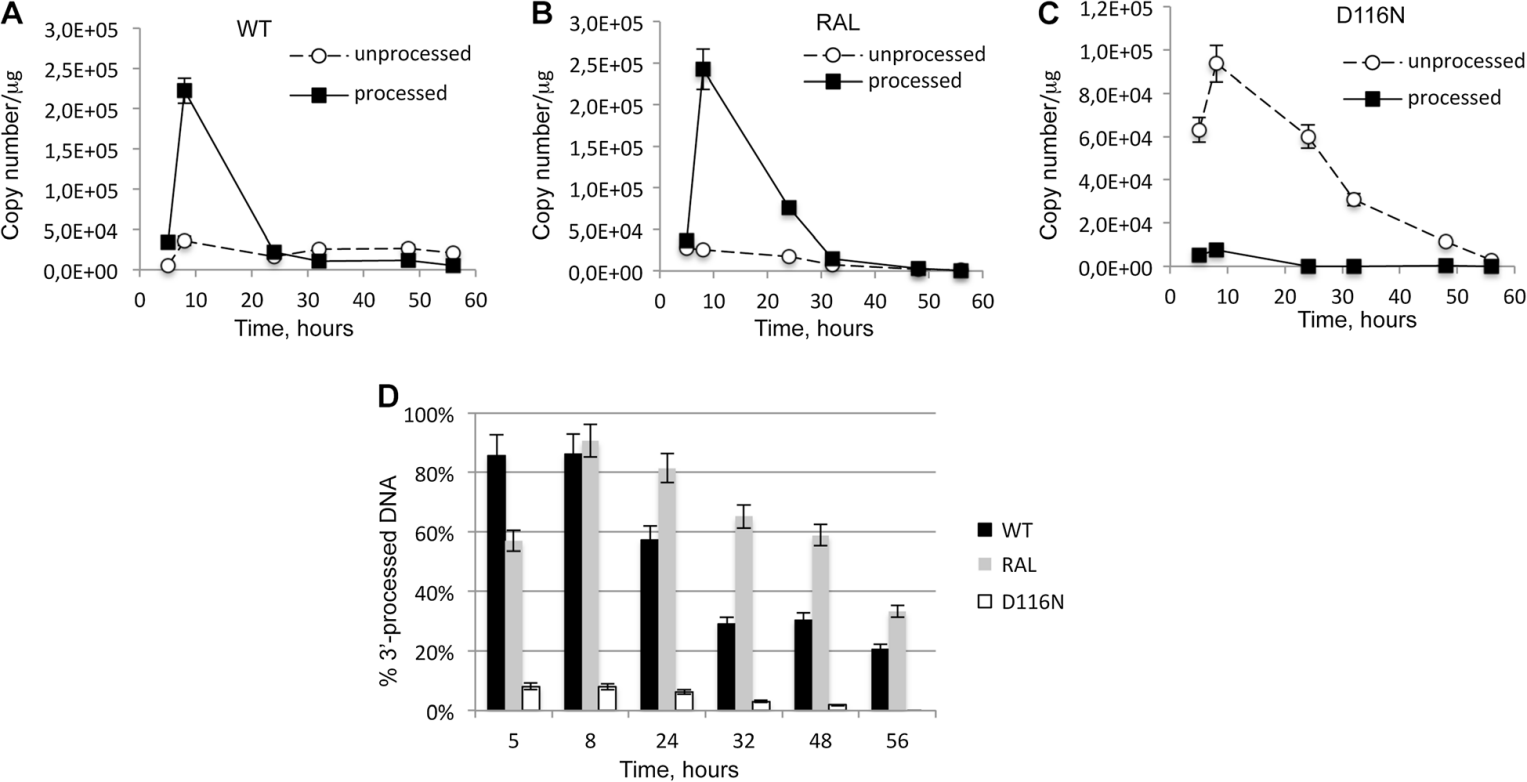
**Efficiency of the 3′-processing reaction during a single round of replication.** MT4 cells were infected with the VSV-G-pseudotyped NLENG1-ES-IRES WT (+/- RAL 500 nM) or NLENG1-ES-IRES D116N viruses. **(A)** WT; **(B)** WT + 500 nM RAL; **(C)** D116N: unprocessed linear DNA (uDNA_L_, white circles); 3′-processed linear DNA (pDNA_L_, black squares). **(D)** Percentage of pDNA_L_ (pDNA_L_ over DNA_L_) during the time course of infection (WT: black columns, WT + 500 nM RAL: grey columns, D116N: white columns). Each value corresponds to an average of five to six independent experiments and confidence intervals analysis are shown for a p value <0.05.

Both the D116N mutation and RAL treatment prevent integration; however, only D116N is expected to prevent the pDNA_L_ formation [7,39]. Indeed, RAL, like other strand transfer inhibitors, is relatively ineffective against the 3′-processing reaction. Accordingly, no significant amount of pDNA_L_ was detected with D116N (Figure 6C). In contrast, there was no significant difference between the 3′-processing activity for WT + RAL (500 nM) and WT without RAL (Figure 6A and Figure 6B, respectively). Moreover, the 3′-processing reaction was concomitant with reverse transcription as the maximum amounts of both linear viral DNA (Figure 3G) and pDNA_L_ (Figure 6A) were found at 8 h p.i.. The reaction yield for the 3′-processing reaction was high (more than 80% of DNA_L_ was processed at 8 h p.i.) and corresponds to a fast reaction as the times characterizing the maximum amounts of total linear viral DNA and pDNA_L_ coincide according to Miller’s study [40]. The pDNA_L_:(pDNA_L_ + uDNA_L_) ratio decreased with time after 8 h p.i. in all experimental conditions (WT+/-RAL) indicating that pDNA_L_ was less stable than uDNA_L_ (Figure 6D). For each time point along the decreasing phase, this ratio was consistently lower in the absence of RAL than presence of RAL. Such a difference most likely accounts for integration events that occur in the absence of RAL.

### Differential inhibition of the 3′-processing reaction by RAL, EVG and DTG

**Figure 7:**
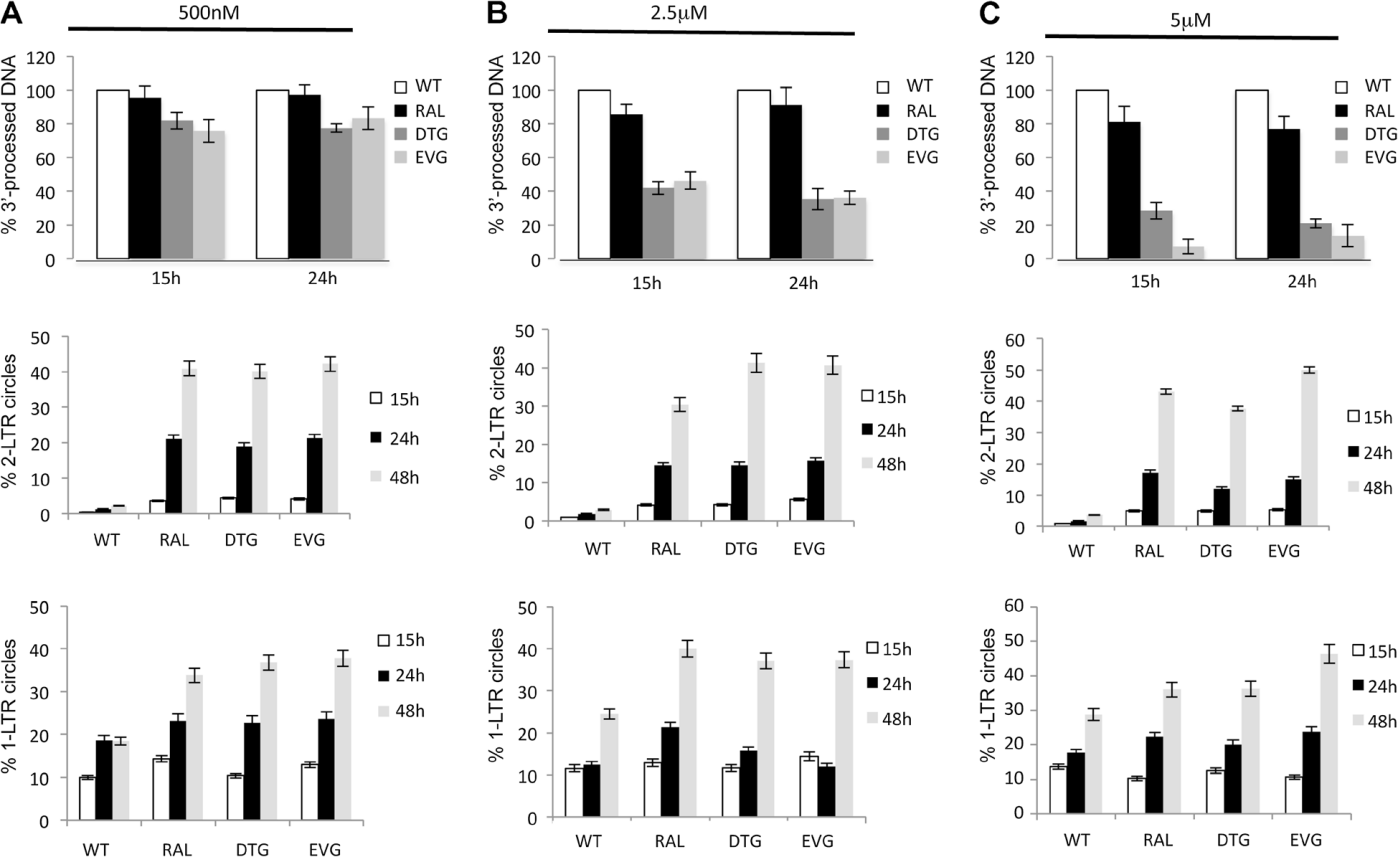
**Strand-transfer inhibitors have different effects on the 3′-processing reaction.** MT4 cells were infected with VSV-G-pseudotyped NLENG1-ES-IRES WT virus in the absence or in the presence of increasing concentration of strand-transfer inhibitors (RAL, DTG or EVG): 500 nM **(A)**, 2.5 μM **(B)** or 5 μM **(C)**. Viral DNA was analyzed 15 h and 24 h post-infection. The percentage of the 3′-processed DNA was normalized to the value obtained in the WT condition (upper panels). WT: white columns; RAL: black columns; DTG: dark grey squares; EVG: grey columns. Percentages of 2-LTRc and 1-LTRc are shown in middle panels and bottom panels, respectively. Each value corresponds to an average of five to six independent experiments and confidence intervals analysis are shown for a p value <0.05.

Two other strand-transfer inhibitors, DTG and EVG [41,42], were studied for their ability to inhibit the 3′-processing reaction and were systematically compared to RAL. The three drugs display similar IC_50_ for the integration inhibition during viral infection: 2 nM for DTG and EVG and 8 nM for RAL (Additional file 1: Figure S4A). Quantitative PCR was used to assay pDNA_L_ and uDNA_L_ as described above. At the lowest concentration (500 nM) of RAL, DTG or EVG, which fully inhibits viral integration, the 3′-processing step was not significantly impaired (Figure 7A, upper panel). Interestingly, the three drugs at higher concentrations had different effects on the 3′-processing reaction (Figure 7B-C, upper panels). Indeed, RAL at concentrations up to 5 μM did not effectively inhibit 3′-processing (3′-processing efficiency was still 80% at 5 μM RAL). By contrast, 3′-processing was greatly impaired using 2.5 or 5 μM of DTG and EVG (3′-processing efficiencies were about 40% and below 25% at 2.5 μM and 5 μM, respectively). Accordingly, *in vitro* studies, using recombinant integrase, demonstrate that the three integrase inhibitors do not inhibit the 3′-processing reaction to the same extent (Additional file 1: Figure S4B). IC_50_ values for 3′-processing inhibition were 2 μM, 5 μM and 10 μM for DTG, EVG and RAL, respectively according to Metifiot et al. study for RAL and EVG [43]. The differential effects of the three compounds on the 3′-processing reaction were confirmed using a radiolabeled probe produced by PCR (as described in the online methods section) (Additional file 1: Figure S4C). As shown in Figure 7, the presence of 500 nM of any of RAL, DTG and EVG led to similar amounts of 3′-processed DNA whereas, at 5 μM, DTG and EVG significantly inhibited 3′-processing, in contrast to that observed for RAL.

Interestingly, increasing the DTG or EVG concentration, from 500 nM to 5 μM, progressively increased the inhibition of 3′-processing but did not lead to a greater accumulation of 2-LTRc (Figure 7A-C, middle and lower panels). Indeed, across this concentration range where DTG and EVG equally (and fully) inhibit integration while 3′-processing inhibition is concentration dependent. 2-LTRc consistently represent nearly 40% of the total viral DNA 48 h p.i.. Such a comparable global accumulation of 2-LTRc, regardless of the 3′-processing efficiency, could be explained as above-mentioned by a different proportion of 2-LTRc harboring perfect/imperfect junction which is modulated by the 3′-processing efficiency (see Figure 4). All together, our data show that, at submicromolar concentration, INSTIs behave similarly in the virological context and *in vitro* (i.e. they are specific inhibitors of the strand-transfer reaction and have little effect on the 3′-processing reaction), and that the 3′-processing reaction influences the nature of 2-LTRc qualitatively but not quantitatively. We also measured 3′-processing efficiency during infection of primary CD4 + T cells with the previously described viruses Δenv WT (+/- 5 μM RAL, EVG or DTG). As described previously during MT4 infection, 3′-processing reaction efficiency was high (75%) but slightly delayed in primary cells (3′-processing reaction peaked 24 h p.i.) and was influenced by 5 μM EVG and DTG but not RAL, as previously found in MT4 cells (Additional file 1: Figure S5). Furthermore, the kinetics of 2-LTRc and 1-LTRc were similar in primary cells and in MT4 infections (Additional file 1: Figure S5, middle and right panels).

## Discussion

Methods are available to quantify accurately various HIV-1 viral DNA forms including total viral DNA, 2-LTRc and integrated viral DNA [18]. However, no methodology had been developed for assaying 1-LTRc in a sensitive manner. It has even been suggested that PCR-based methods are unable to quantify 1-LTRc [20]. Amplification with primers spanning the LTR leads to unspecific amplification from both DNA_L_ and 2-LTRc. The appropriate application of one crucial parameter (elongation time) explains why our methodology is accurate for 1-LTRc quantification. We found empirically that an elongation time of 25 s was optimal for efficient and specific amplification of 1-LTRc. A shorter elongation time (18 s) was associated with a lower amplification efficiency whereas a longer elongation time (32 s) resulted in unspecific amplification from DNA mimicking DNA_L_ (Additional file 1: Figure S2). For example, amplification of 200,000 copies of DNA_L_ (as quantified by primers and probes used for total viral DNA quantification) using our 1-LTRc protocol, but with an elongation time of 32 s, led to nearly 87,000 copies corresponding to 43% of unspecific amplification (Additional file 1: Figure S2, right table). Our results, sustained by those obtained by Yoder and colleagues, indicate that increasing elongation time leads to unspecific amplification and particularly from DNA_L_. The elongation time used by Yoder and colleagues was even longer (60s), and not compatible with a reliable quantification of 1-LTRc [20]. Concerning DNA_L_, the number of cycles in the first PCR (12 cycles) was determinant for reliable quantification. We compared the sensitivity of our method to that of Southern blotting: the sensitivity of our quantitative PCR was 10^2^ copies/10^6^ cells, whereas that of Southern blotting was 10^5^ copies/10^6^ cells.

We used these methods to determine the amounts of 1-LTRc and DNA_L_ in its two forms (3′-processed DNA (pDNA_L_) or unprocessed (uDNA_L_)) in several conditions of infections. The findings of these analyses contribute to understanding various unresolved issues (i) the precise timing, efficiency and localization of the processing step required for the proper integration of viral DNA (ii) the relative lifetimes of DNA_L_ and circular viral forms (iii) the mechanism of action of anti-integrase compounds during infection (*i*.*e*. inhibitors of 3′-processing only, inhibitors of strand-transfer only or inhibitors of both reactions) and (iv) the localization of the formation of circular viral DNA forms.

### Characterization of linear viral DNA forms

Regarding the quantification of DNA_L_, our quantitative PCR-based methodology allows to discriminate between uDNA_L_ and pDNA_L_. We showed that the 3′-processing reaction corresponds to an early event that is concomitant with the reverse transcription step, evidence that 3′-processing occurs in the cytoplasmic compartment. In both MT4 cell line and primary CD4 + T cells, 85% of the DNA_L_ is 3′-processed (Figure 6D) indicating that 3′-processing in the wild-type context is efficient. Given this high reaction efficiency, it is unlikely that 3′-processing is the limiting factor for integration (40% of the total viral DNA is integrated (Figure 3B)). The relatively low integration yield may be a consequence of the formation of circular forms, poor stability of pDNA_L_ (see below) or inefficiency of the strand transfer reaction.

We found the following order for the stability of the viral DNA forms: integrated DNA > circular DNA (1-LTRc and 2-LTRc) > uDNA_L_ > pDNA_L_. Integrated DNA is the most stable form due to its replication with cellular DNA, whereas circular forms are diluted by cell division. The loss of DNA_L_ is believed to be the consequence of several phenomena: integration of pDNA_L_, degradation of viral extremities by cellular proteins and circularization of DNA_L_[44-46]. Additionally, we found that the lifetime of pDNA_L_ was shorter compared to uDNA_L_ and therefore that pDNA_L_ is intrinsically less stable than uDNA_L_. Indeed, in the absence of RAL, pDNA_L_ is integrated into the host cell genome, explaining, at least in part, its disappearance. However, even in the presence of RAL such that no pDNA_L_ is integrated, pDNA_L_ remains less stable than uDNA_L_. The relative instability of pDNA_L_ in this condition is probably due to a conformational modification of IN after 3′-processing resulting in a lower stability of IN itself on the viral DNA end. In the absence of any integration event, IN can dissociate from the LTR ends leading to degradation of the LTR ends.

Nanomolar concentrations of RAL, DTG and EVG efficiently inhibit the integration process [47,48]. We found that, although RAL, DTG and EVG in the micromolar range inhibit 3′-processing to different extents both *in vitro* and in the virological context, none of these compounds inhibit 3′-processing in infected cells at submicromolar concentrations that fully inhibit integration (*e*.*g*. 500 nM). This demonstrates that RAL, DTG and EVG are primarily INSTI compounds in the virological context. As uDNA_L_ is more stable than pDNA_L_, it is important to know the performance of anti-IN inhibitors, with clinical potential, against 3′-processing in the virological context. Indeed, compounds which, in the same concentration range, inhibit both integration and 3′-processing reactions, may favor accumulation of uDNA_L_ relative to pDNA_L_ (due to 3′-processing inhibition). Due to the greater stability of uDNA_L_, this could be a risk factor for viral resumption.

### Formation of circular viral DNA forms

It has been clearly demonstrated that 2-LTRc are formed in the nucleus of infected cells [49], and we confirmed this result (Figure 5). The situation is less clear for 1-LTRc. Indeed, a significant amount of 1-LTRc (10% of total 1-LTRc) was detected in the cytoplasmic fraction, consistent with 1-LTRc formation being linked to reverse transcription as early suggested by Miller and colleagues [30]. However, we found that 90% of the 1-LTRc was in the nuclear compartment, and that the total mount of 1-LTRc was reduced when nuclear import was impaired (using peptides inhibiting the integrase-importinα or a PPT/CTS mutant). Therefore, most of 1-LTRc are generated in the nucleus. Thus, our results reconciles apparent contradictions in the literature and indicate that there are two mechanisms of 1-LTRc formation co-exist in infected cells, whereas 2-LTRc are exclusively formed in the nucleus.

We also observed that the overall amount of 2-LTRc accumulation (including both of 2-LTRc: the first subgroup encompassing a perfect palindromic junction and the second harboring an imperfect palindromic junction) was not directly related to the 3′-processing reaction: increasing EVG or DTG concentrations from 500 nM to 5 μM, and thereby modulating the 3′-processing reaction yield, did not affect the 2-LTRc accumulation. This confirms previous studies reporting that amount of 2-LTRc was the same after RAL treatment (believed not to inhibit 3′-processing) or infection with the D116N mutant (3′-processing is inhibited) [29,50]. This result is somewhat surprising since, due to the incompatibility of the 3′-processed ends with circularization of DNA_L_, the amount of 2-LTRc would be expected to be impaired. However, we found that 500 nM RAL did not inhibit 3′-processing in the virological context or *in vitro*, and that RAL treatment and D116N did not led to accumulation of the two 2-LTRc subgroups to the same extent. Following RAL treatment, about 50% of 2-LTRc have a perfect palindromic junction, as in the case in control WT conditions, whereas in the D116N condition 80% of the 2-LTRc have a perfect palindromic junction (Figure 4). In conclusion, 3′-processing efficiency does not influence the total amount of 2-LTRc but affects the nature of the palindromic junction in the 2-LTRc (perfect versus imperfect junctions).

## Conclusions

Our methods allowing accurate quantification of 1-LTRc and DNA_L_ have provided important information about the fate of the various viral DNA forms (integrated viral DNA, 2-LTRc, 1-LTRc and DNA_L_) during viral infection and could be applied to study the lifetime of circular and linear DNA in patients. Other viral DNA forms originating from auto-integration exist. As underlined by Yan et al., it is a hard task to quantify these viral DNA forms due to their heterogeneous nature [15]. However, these forms appear to be not highly represented relative to the total viral DNA. Indeed, our data show that the amount of total viral DNA is similar to the addition of integrated, 1-LTRc, 2-LTRc and DNA_L_ amounts. These methods can be used to follow the fate of viral DNA forms, the distributions of which may be influenced by mutations or inhibitors of HIV-1 viral proteins. Indeed, the quantification of 3′-processed DNA in infected cells may help to elucidate, directly in the cell context, the mechanism of integrase inhibitors developed for clinical applications.

## Methods

### Cells and viruses

MT4 and Nalm6/Nalm114 cells were cultured in RPMI1640. The ligase 4 gene was knockout from the parental cell line Nalm-6 to obtain Nalm-114 cells. Ligase 4 is a component of the NHEJ (Non-Homologous End Joining) pathway involved in 2-LTRc formation [12]. HeLa and 293 T cells were cultured in DMEM. Both mediums were supplemented with 10% fetal calf serum. HIV-1 pNL4.3 stocks were prepared by transfecting 293 T with the HIV-1 molecular clone pNL4.3 or with HIV-1 molecular clones derived from the pNL4-3 (Δenv viruses) [26]. Δenv viruses NLENG1-ES-IRES WT and NLENG1-ES-IRES D116N encode the WT and catalytically inactive mutant D116N, respectively. Pseudotyping of Δenv viruses was performed by co-transfection of 293 T cells with a VSV-G plasmid using the calcium phosphate method. Viral supernatants were filtered (0.45 μm) and frozen at −80°C.

### Isolation of highly purified CD4 + T cells

Highly purified CD4 + T cells were isolated from peripheral blood mononuclear cells (PBMC) of HIV-1 negative donors from EFS (Etablissement Français du Sang). Briefly, PBMC were obtained by centrifugation on Ficoll-Hypaque gradient. Purification of CD4 + T cells was achieved by staining cells with CD4 MicroBeads (MACS®, Miltenyi Biotec) and purified with the Whole Blood Column Kit (MACS®, Miltenyi Biotec). Purified CD4 + T cells were cultured in RPMI1640 supplemented with 2% Human serum, penicillin-streptomicine, and in presence of IL-2 (50 ng/ml). CD4 + T cells were activated with phytohemagglutinin (PHA, 2.5 μg/ml) during 3 days and were used for experiments 7 days after the activation treatment.

### Viral infection

HIV-1 p24^gag^ antigen contents in viral inocula were determined by enzyme-linked immunosorbent assay (Perkin-Elmer Life Sciences). For the WT, 120 ng of p24^gag^ antigen per 10^6^ cells, corresponding to a multiplicity of infection (m.o.i.) of 0.3, was used for infection. Primary CD4 + T cells were infected with 100 ng of p24^gag^ antigen per 10^6^ cells. When required, cells were treated in the presence of several integrase inhibitors such as RAL, DTG and EVG at 500 nM, 2.5 μM or 5 μM. Two to five millions cells were collected at each time point. Cells were washed in PBS, and dry cell pellets were frozen at -80°C until use. DNA from infected cells was purified with QIAamp DNA Blood mini kit (Qiagen) according to the manufacturer’s instructions. To digest residual transfection plasmid, DNA was incubated with 10 units of DpnI (NEB) according to the manufacturer’s instructions for 4 hours at 37°C.

### Plasmids

Four plasmids were constructed for standard curves amplification: p1-LTR, p2-LTR, pLIN-HIV-ScaI and pLIN-HIV-NdeI.

pLIN-HIV-ScaI plasmid was constructed using a linker-mediated PCR (LM-PCR). MT4 cells were infected with pNL4.3 HIV-1 and DNA was extracted. The three terminal nucleotides of HIV-1 DNA LTR represent a half of the ScaI restriction site. Viral DNA was ligated with a linker composed of oligonucleotides 25SCAt (5′-GCGGTGACCCGGGAGATCTGAATTCAGT-3′) and 11SCAb (5′-ACTGAATTCAGATCTCCCGG-3′), containing the complementary moiety of ScaI site. The ligation product was next used to amplify the termini of linear viral genome linked with the linker by PCR using primers 25 t and MS1 (see Additional file 1: Table S1B). PCR was performed as follows: 95°C/30 sec, 55°C/30 sec and 68°C/1mn for 35 cycles. The reaction product was purified on agarose gel, cloned into the pGEM-T easy vectors (Promega) and sequenced. Note that the ScaI site in the pGEM-T easy vector was removed by site directed mutagenesis (QuikChange Lightning Kits, Agilent). Site directed mutagenesis (QuikChange Lightning Kits, Agilent) was also performed on this plasmid to remove the ScaI recognition site in position 314 in the LTR5′ with primers 5′-CCCGAGAGCTGCATCCGGAGAACTACAAAGACTGCTGACATCG-3′ and 5′-CGATGTCAGCAGTCTTTGTAGTTCTCCGGATGCAGCTCTCGGG-3′. The final plasmid, pLIN-HIV-ScaI, contains only one ScaI site at the bounder of the linker and LTR5′ extremity. Digestion with ScaI and AatII leads to a fragment mimicking the extremity (LTR5′) of unprocessed viral DNA end. After purification, this fragment was used for ligation reaction with linker 11b in order to quantify unprocessed linear DNA (uDNA_L_).

pLIN-HIV-NdeI was constructed by site directed mutagenesis (QuikChange Lightning Kits, Agilent) to replace the ScaI site by NdeI site at the linker-viral DNA junction, using primers 5′-CCGGGAGATCTGAATTCAGTCATATGGAAGGGCTAATTTGGTCC-3′ and 5′-GGACCAAA TTAGCCCTTCCATATGACTGAATTCAGATCTCCCGG-3′. The digestion with NdeI and AatII leads to a fragment mimicking the extremity of the 3′-processed viral DNA. After purification, this fragment was used for ligation reaction with linker 11TAb.

p1-LTR was obtained by amplification, from HIV-1 infected cells DNA, of the *env*-LTR-*gag* region, specifically present on 1-LTRc, using primers 1LTR LA1 and 1LTR LA16 (see Additional file 1: Table S1B). This amplification product was cloned into the pGEMT-easy vector (Promega) to give p1-LTR.

p2-LTR was constructed in two steps as follows: pLIN-HIV-ScaI-LTR3′ was first constructed by the same methodology described for pLIN-HIV-ScaI. Primers used for LM-PCR were 25 t and 1LTR LA16 (see Additional file 1: Table S1B), resulting in amplification of the *env*-LTR3′ region. The ScaI recognition site present in the LTR3′ of pLIN-HIV-ScaI-LTR3′ was mutated (as previously done for the ScaI site in the LTR5′ of pLIN-HIV-ScaI) by site directed mutagenesis (QuikChange Lightning Kits, Agilent) using primers 5′-AGCTGCATCCGGAGCACTTCAAGAACTGCT-3′ and 5′-AGCAGTTCTTGAAGTGCTCCGGATGCAGCT-3′. The two plasmids, pLIN-HIV-ScaI and pLIN-HIV-ScaI-LTR3′, were digested by ScaI, and fragments containing respectively the *env*-LTR3′ and the *gag*-LTR5′ regions were purified on agarose gel and ligated together into the pGEMT-easy vector (Promega) to give p-2LTR.

### Quantification of total linear DNA (DNA_L_), unprocessed (uDNA_L_) and 3′-processed (pDNA_L_) linear forms by LM-PCR

#### Principle

DNA_L_ quantification was performed by a linker-mediated PCR approach (LM-PCR). The choice of the linkers was based on the early study by Pierson and colleagues [22]. In order to quantify either the total amount of linear DNA (DNA_L_) -which comprises both the unprocessed (uDNA_L_) and the 3′-processed (pDNA_L_)- or, more specifically, the uDNA_L_ only, we used the linkers 11TAb and 11b, respectively, for establishing standard calibration (Additional file 1: Table S1A). Two rounds of PCR were performed using primers and probes described in Additional file 1: Table S1B; the number of rounds of the first PCR was critical for further quantitative analysis and we found that 12 cycles correspond to the optimal condition for the two linkers (Additional file 1: Figure S6).

#### Method

Quantifications were performed by real-time PCR on a Light cycler instrument (Roche Diagnostics) using the second-derivative-maximum method provided by the Light Cycler quantification software, version 3.5 (Roche Diagnostics). Two linkers: Linker 11b and 11TAb (or 11GTb in the virological context; see below) (see Additional file 1: Table S1A) were used for the quantification of uDNA_L_ and pDNA_L_, respectively. These linkers were assembled by annealing two partially complementary unphosphorylated oligonucleotides (to give 34 nM) final concentration) in the presence of 200 mM NaCl. To quantify linear forms of HIV-1 DNA, a ligation reaction mixture was carried out by addition of linkers (final concentration: 30 nM) to DNA, in the presence of 10 units of ligase from the Quick ligation kit (NEB), for 2 hours at room temperature in a final volume of 20 μL, according to the manufacturer’s instructions. Linked DNA products were then purified with PCR switch charge purification kits (Life Technology) according to the manufacturer’s instructions (to prevent inhibition of the PCR due to the mixture of ligation reaction) and eluted in 20 μL and then submitted to real-time PCR. In a first round of PCR, 1/10 of DNA was amplified in duplicate in a 20 μl reaction mixture comprising 1 × LightCycler FastStart DNA master Hybprobes (Roche), 4 mM MgCl_2_, 32 t and MS1 primers (300nM) for quantification of the LTR5′ (Additional file 1: Table S1B) or 32 t and 1LTR LA15 (5′- CACACCTCAGGTACCTTTAAGA-3′) (300 nM) for LTR3′. To remain in the exponential phase allowing quantitative properties of the second PCR, 12 cycles are required for the first PCR. Decreasing the number of cycles for the first PCR leads a non-reproducibility in the samples quantifications (Additional file 1: Figure S6). Increasing the number of cycles for the first PCR results in a false quantification because the exponential phase allowing quantitative properties of the second PCR is not respected (Additional file 1: Figure S6). 12 cycles are sufficient to ensure quantitative conditions for all linked-DNA dilutions for the second PCR. The second PCR was performed on 1/100 of the first PCR-product in a mixture comprising 1 × LightCycler FastStart DNA master Hybprobes, 4 mM MgCl_2_, 25 t and MS2 primers (300 nM) and hybridization probes MH FL and MH LC (200 nM) for quantification of the LTR5′ (Additional file 1: Table S1B) or 25 t and 1LTRnested (5′- GCTAATTCACTCCCAACGAAG-3′) (300 nM) and hybridization probes LTR FL and LTR LC (200 nM) (Additional file 1: Table S1B) for LTR3′. Efficiency of the uDNA_L_ quantification was determined by addition of the linker 11b to serial dilutions of the fragment from the digestion of pLIN-HIV-ScaI with ScaI and AatII. For pDNA_L_ quantification, the efficiency of the procedure was determined by addition of the linker 11TAb (composed of oligonucleotides 25 t and 11TAb) (see Additional file 1: Table S1A) to serial dilutions of the fragment obtained after digestion of pLIN-HIV-NdeI with NdeI and AatII. 11GTb characterization as well as comparison between Southern blot and quantitative PCR were shown in Additional file 1: Figure S1).

### Method validation

To assess both the sensitivity and the linear range of amplification, we used DNA mimicking the uDNA_L_ or pDNA_L_ (obtained by ScaI/AatII or NdeI/AatII digestion of pLIN-HIV-ScaI or pLIN-HIV-NdeI, respectively (Figure 1A)). uDNA_L_ or pDNA_L_ were quantified independently using total viral DNA quantification protocol (line 1, Additional file 1: Table S1B). The standard curves were monitored by serial dilutions of the fragments mimicking uDNA_L_ or pDNA_L_ in DNA of uninfected cells. Linkers 11TAb or 11b were used for ligation of each dilutions of viral DNA extremity (uDNA_L_ or pDNA_L_). After ligation and DNA purification, 1/10 of the ligation reaction was submitted to real-time PCR (12 cycles as above-mentioned). It is important to note that quantifications of samples account for the ligation efficiency. Amplified products were diluted (1/10) and next submitted to the second PCR round. Efficiencies and sensitivities related to uDNA_L_ and pDNA_L_ quantification were identical (90% efficiency on a 7-log range; sensitivity of 10 copies for 200,000 cells) (Figure 1B). To assess the specificity of the quantification procedure, we tested the detection efficiency of pDNA_L_ or uDNA_L_ when using either 11TAb or 11b linker (Figure 1C). We confirmed qualitative results from Pierson [22], *i*.*e*. 11TAb was not able to discriminate between pDNA_L_ and uDNA_L_, while 11b led to detection of uDNA_L_ only. From a quantitative point of view, the detection efficiency of pDNA_L_ and uDNA_L_ by LM-PCR using 11TAb as a linker was identical and high (90%) (Figures 1B and Figure 1C). The LM-PCR with linker 11b allows a high degree of selectivity in the detection, with detection efficiencies of 95% and 3% for uDNA_L_ and pDNA_L_, respectively (Figure 1C). Performing two independent experiments (each one with a different linker), accurate quantifications of the total amount of DNA_L_ and the amount of uDNA_L_ are thus possible. The pDNA_L_ amount can be then simply deduced by subtraction: total DNA_L_ (using 11TAb) minus uDNA_L_ (using 11b). The ability of the linker 11TAb to detect uDNA_L_ could be due to the fact that the overhanging nucleotides of the linker (AT-5′; complementary to the overhanging 5′-TA of pDNA_L_) are not involved in ligation with the phosphate at the 5′-DNA_L_ end, leading to equivalent detection of uDNA_L_ and pDNA_L_.

### Analysis of the time course of 3′-processing reaction in infected cells

For quantification of pDNA_L_ in infected cells, the linker 11TAb was replaced by the linker 11GTb (the above mentioned linker 11b is still used for uDNA_L_ quantification in infected cells). Ligations for the different standard curves were performed in uninfected cells DNA (200 ng/μl) in order to check that the ligation/amplification efficiency was not influenced by the trapping of linker by uninfected DNA. The copy number of linear DNA was determined in reference to a standard curve prepared by amplification of quantities ranging from 10 to 10^5^ copies of corresponding digested fragments. PCR parameters for all PCR protocols are given in Additional file 1: Table S1B. We demonstrated that the 11GTb linker is able to detect the uDNA_L_ and the pDNA_L_ with a similar efficiency (90%) (Figure 1B), whereas the linker 11b can only detect uDNA_L_. These two parameters have been taking into account for the calculation of pDNA_L_ amount. Formula given the amounts of unprocessed DNA_L_ (uDNA_L_) and 3′-processed DNA_L_ (pDNA_L_) are described below:

uDNA_L_ = amount found with the linker 11b,

pDNA_L_ = amount found with the linker 11GTb - amount found with the linker 11b;

If no processing occurs (see below) the values obtained with linkers 11b and 11GTb are identical.

### Quantification of 1-LTR circles

#### Principle

One problem of 1-LTRc quantification is that primers hybridizing in the *env* and *gag* genes could lead to amplification of the LTR-LTR region present in 2-LTRc and amplification of DNA_L_ via LTR recombination (see Figure 2A). In the method described below, we established PCR condition (mainly the elongation time of the PCR) which leads to specific detection of 1-LTRc.

### Method and validation

For 1-LTRc quantification, reaction mixture contained 1 × LightCycler FastStart DNA master Hybprobes (Roche Diagnostics), 4 mM MgCl_2_, 300 nM of primers, and hybridization probes (200 nM each), in a final volume of 20 μl. PCR cycle conditions are shown in Additional file 1: Table S1B. Optimal elongation time for further quantitative analysis was found to be 25 s. Amplification using 1LTR LA1 and 1LTR LA16 (Additional file 1: Table S1B) was performed with p1-LTR (Figure 2B) used as a standard curve. p2-LTR (Figure 2B) which contains two full-length LTRs flanked by the *gag* and *env* genes was used as a control. Quantitative PCR using p1-LTR led to high amplification (92.5-100%) and sensitive detection (200 copies/10^6^ cells) of 1-LTRc (Figure 2 C1). Under the same condition, p2-LTR led to weak amplification (0.7-2%), regardless of the initial amount used (Figure 2 C2). Next, p2-LTR was digested using ScaI to mimic DNA_L_ (Figure 2B). As found for p2-LTR, DNA_L_ amplifications was found to be negligible (<0.1%) (Figure 2 C3). Taken together, our results show that our protocol is compatible with an accurate 1-LTRc quantification and overcomes the bias due to DNA_L_ and/or 2-LTRc amplification.

Quantifications of 2-LTR circles harboring perfect or imperfect LTR-LTR junction, total HIV-1 DNA, integrated viral DNA, ß-globin gene and mitochondrial 12S gene.

These real-time PCR quantifications were based on well established protocols. Sequences of primers and probes for 2-LTR circles, total HIV-1 DNA and integrated viral DNA are given in Additional file 1: Table S1B. Briefly, for 2-LTRc and total viral DNA quantifications, reaction mixtures contained 1 × LightCycler FastStart DNA master Hybprobes (Roche Diagnostics), 4 mM MgCl_2_, 300 nM of primers, and hybridization probes (200 nM each), in a final volume of 20 μl. PCR cycle conditions are shown in Additional file 1: Table S1B. Copy numbers of the different forms of viral DNA were determined in reference to a standard curve prepared by serial dilutions of the corresponding plasmid: p2-LTR and pNL4.3 for 2-LTRc and total viral DNA quantifications, respectively. Quantification of 2-LTRc harboring a perfect LTR-LTR junction has been achieved according to De Iaco and colleagues [11]. Briefly, 2-LTRc harboring a perfect LTR-LTR junction were quantified using HIV-R1: 5′-ACTGGTACTAGCTTGTAGCACCATCCA-3′, a primer overlapping the perfect 2-LTRc junction Junct4-fwd: 5′- CAGTGTGGAAAATCTCTAGCAGTACTG-3′ and two fluorogenic hybridization probes HIV-FL: 5′-CCACACACAAGGCTACTTCCCTGA-3′ and HIV-LC: 5′-TGGCAGAACTACACACCAGGGC-3′. Reaction mixtures contained 1 × Light Cycler Fast Start DNA master hybridization probes (Roche Diagnostics), 4 mM MgCl2, 300 nM forward and reverse primers, and 200 nM (each) fluorogenic hybridization probe, in a final volume of 20 μl. PCR cycle conditions for conventional and perfect two-LTR circles HIV-1 DNA amplifications were (denaturation: 95°C, 8 min; PCR cycles: 95°C, 10 s, 60°C, 10 s, 72°C, 6 s for 50 cycles). Quantification of integrated viral DNA was performed as described previously [29]. Human ß-globin gene was quantified with commercially available materials (Control kit DNA; Roche Diagnostics). The mitochondrial 12S gene was quantified using the protocol developed by Petit and colleagues [51].

### 3′-processing of U5 extremity with radiolabeled probes

MT4 (5.10^6^ cells) were infected with VSV-G-pseudotyped NLENG1-ES-IRES D116N or NLENG1-ES-IRES WT +/- RAL, DTG or EVG (500 nM or 5 μM). 10 h post-infection, DNA was extracted from the cytoplasmic compartment and digested with HindIII. The digested DNA was fractionated through DNA sequencing gels. After electrophoresis, DNA was transferred on a Hybond-N + membrane (Amersham) according to manufacturer’s instructions. For detection of both unprocessed and processed U5 extremity, a PCR fragment was produced with 5′-GTGCCCGTCTGTTGTGTGACT-3′ and 5′-ACTGGTACTAGCTTGTAGCACCATCCA-3′ primers in the presence of α-CTP^32^. After purification, this PCR probe was heated (95°C, 5 min) and used for hydridization of the membrane according to the manufacturer’s instructions. Then, the membrane was washed and processed for autoradiography. Southern blot in Figure S1A has been performed with DNA from MT4 cells infected with NLENG1-ES-IRES D116N. Briefly, DNA was extracted, digested with SpeI, purified and quantified using the LM-PCR and total viral DNA protocols. DNA was loaded on a 1% agarose gel and detection was performed using the PCR probe described above.

### Cell fractionation

5.10^6^ cells were infected with VSV-G-pseudotyped NLENG1-ES-IRES D116N. 24 h post infection, cells were harvested, washed with PBS and the pellet was resuspended in 0.5 mL of isotonic buffer 1 (20 mM HEPES pH 7.4, 110 mM KCl, 5 mM MgCl_2_, 0.5 mM EGTA, 1 mM DTT, 20 μg/ml aprotinin, 20 μg/ml leupeptin). Samples were centrifuged for 2 min at 420 g (4°C); the pellet was gently resuspended on ice in 50 μl of isotonic buffer 1, then 0.5 ml isotonic buffer + 0,005% digitonine was added and samples were incubated for 5 min on ice. Following a 2 min centrifugation (420 g at 4°C), the supernatant was cleared by centrifugation at 8,600 g (20 min at 4°C). The supernatant constitutes the cytoplasmic compartment. The pellet was washed in 0.5 ml of isotonic buffer 1 once, resuspended in 1 ml of isotonic buffer 2 (50 mM TrisHCl pH 7.5, 25 mM KCl, 5 mM MgCl_2_, 0.25 M sucrose) mixed with 2 ml of isotonic buffer 2 + 2.3 M sucrose and placed in a 5 ml ultracentrifuge tube on ice. Samples were then underlayed with 1 ml of isotonic buffer 2 + 2.3 M sucrose and centrifuged at 88,000 g in a SW55 TI rotor at 4°C for 40 min. The interface containing purified nuclei was collected. Nuclei and cytoplasmic extracts were mixed with 1 volume of 2 × lysis buffer (100 mM Tris-HCl pH 8, 1% SDS, 10 mM EDTA, 50 μg/ml proteinase K), incubated for 4 hours at 55°C; nucleic acids were isolated by phenol/chloroform and ethanol precipitation.

### Peptides

Peptides used in this study (NLS-IN-Pen and SV40-NLS-Pen) were previously described to inhibit HIV-1 integrase nuclear import [32]. They were purchased from GeneCust at >95% purity. HeLa cells were growth, arrested with 5 μg/ml of aphidicholine and then incubated with 100 μM of peptide for 6 h. Cells were then infected as above described. Viral DNA molecules were then analyzed by quantitative PCR.

### Mutant CTS/PPT

The viral molecular clone, kindly provided by Dr Nathalie Arhel, used in this study is described to be impaired in the nuclear import (cPPT and CTS double mutant) due to the disruption of the FLAP structure [23].

### Characterization of integrase enzymatic activity *in vitro*

Recombinant Integrase was produced in *Escherichia coli* BL21-CodonPlus (DE3)RIPL (Agilent, Santa Clara, USA) and purified under non-denaturing conditions, as previously described [52]. Oligonucleotide (ODN) mimicking the U5 LTR end of the viral genome (U5B) was radiolabeled with T4 polynucleotide kinase (Biolabs, Ipswich, USA) and [γ-^32^P] ATP (Amersham, GE Healthcare, USA), then purified on a Sephadex G-10 column. Double-stranded ODN was obtained by mixing equimolar amount of complementary strand in the presence of 100 mM NaCl. 3′-processing assay was carried out at 37°C in a buffer containing 20 mM HEPES (pH 6.8), 1 mM dithiothreitol (DTT), 7.5 mM MgCl_2_ and 50 mM NaCl in the presence of a 6.25 nM U5A/U5B double-stranded DNA substrate. Products were separated by in a 16% acrylamide/urea denaturing gel, analyzed with a Typhoon TRIO variable mode imager (GE Healthcare, USA) and quantified with ImageQuant TL software. The susceptibility of IN to RAL, EVG and DTG was determined *in vitro* by assessing IN activity in the presence of various concentrations of strand transfer inhibitors. 50% inhibitory concentrations (IC50) were determined with Prism 5.0 software. The HIV-1 ODN substrate sequences were: U5B: 5′-GTGTGGAAAATCTCTAGCAGT-3′; U5A: 5′-ACTGCTAGAGATTTTCCACAC-3′.

## Competing interests

The authors declare that they have no competing interests.

## Authors’ contributions

Conceived and designed the experiments: SM, ED, OD. Performed the experiments: SM, ST, FS. Analyzed the data: SM, ST, FS, ED, OD. Wrote the paper: ED, OD. All authors read and approved the final manuscript.

## Supplementary Material

Additional file 1 The data sets supporting the results of this article are available: Table S1, Figures S1-S6. Click here for file
