# Supplementary material for: Quantitative analysis of the time-course of viral DNA forms during the HIV-1 life cycle
Source: Retrovirology. 2013 Aug 13;10:87. doi: 10.1186/1742-4690-10-87 (PMC3766001; doi:10.1186/1742-4690-10-87)

SUPPLEMENTARY DATA

Table S1: Summary of oligonucleotides and probes used. (**A**) Name, sequences of the oligonucleotides used for the construction of the linkers. (**B**) Name, sequences of the oligonucleotides and probes used for quantitative PCRs. Target and PCR conditions for each quantification are indicated.

Figure S1: Characterization of 11GTb efficiency and Southern blotting sensitivity. (**A**) Southern blotting efficiency. MT4 cells were infected with various amounts of VSV-G-pseudotyped NLENG1-ES-IRES D116N. Viral DNA was digested with SpeI, purified and quantified using total viral DNA and linear viral DNA protocols and then submitted to Southern blotting using radiolabeled probe described in the Methods section. Copy number, determined by quantitative PCR, was indicated. Sensitivity of the quantitative PCR was determined (10^2 copies/10^6 cells). Southern blotting sensitivity was estimated (10^5 copies/10^6 cells). (**B**) Characterization of 11GTb efficiency. As no restriction enzyme is available to mimic the processed viral LTR ends during infection, the efficiency of ligation-amplification was estimated at early time post-infection where total DNA amount is similar to linear DNA with a margin of error of 10% due to the presence of early 1-LTRc (see Figure 3). Total viral DNA was quantified using standard protocol with primers and probes described in Additional file 1: Table S1B. Viral DNA was diluted and submitted to the ligation-amplification protocol using the 11GTb linker. Quantification using the two procedures where in accordance in the margin of error of 10% highlighting that the GT overhang did not strongly influence the ligation efficiency.

Figure S2: 1-LTRc amplification as a function of the elongation time. Determination of the optimal elongation time for 1-LTR circles amplification was determined empirically. An elongation time of 18s leads to a weak 1-LTR circles amplification. An elongation time of 32s leads to an unspecific amplification from 2-LTR circles and linear viral DNA. The optimal elongation time was found to be 25s. %Amplification calculation is based on the output:input ratio.

Figure S3: The 3'-processing reaction is efficient on both LTR extremities. MT4 cells were infected with VSV-G-pseudotyped NLENG1-ES-IRES WT. 8h and 15h post-infection, 3'-processing efficiency was determined on both LTR extremities. The efficiency of the 3'-processing reaction on the LTR3' was determined using the same procedure than the one used for LTR5' with, however, specific primers and probes (see materials and methods section). Each value corresponds to an average of five to six independent experiments and confidence intervals analysis are shown for a p value <0.05.

Figure S4: Inhibition of the 3'-processing reaction by strand transfer inhibitors. (**A**) MT4 cells were infected with WT virus in the presence of increasing concentrations of each compound. Percentage of integrated viral DNA with increasing concentration of RAL (black diamonds), DTG (white circles) and EVG (white squares) was determined 24h post-infection. Efavirenz (EFA; black crosses) was used as a control. (**B**) 3'-processing inhibition of recombinant HIV-1 integrase (purified as previously described, see materials and methods section). HIV-1 integrase was incubated with radiolabeled oligonucleotide mimicking the U5 end of the viral DNA in the presence of increasing concentration of RAL (black diamonds), DTG (white circles) or EVG (white squares). 3'-processing inhibition was quantified according to the protocol described in the online methods section and reported. (**C**) 3'-processing measured in infected cells and analyzed by Southern blot assay. MT4 cells were infected with VSV-G-pseudotyped NLENG1-ES-IRES D116N or NLENG1-ES-IRES WT (+/- RAL, DTG or EVG, at 500nM or 5μM). Efficiency of the 3'-processing as measured for the U5 extremity in the presence of two drug concentrations. The expected sizes of each viral strand after HindIII digestion of processed or unprocessed U5 extremity DNA end are shown besides the autoradiographies.

Figure S5: Quantification of the 3'-processing in infected primary CD4+T cells. Activated CD4+T cells were infected with VSV-G-pseudotyped NLENG1-ES-IRES WT (+/- RAL, DTG or EVG (5μM)). The percentage of 3'-processed DNA over linear DNA was quantified during the time course of infection (left panel); WT: white columns; RAL: black columns; EVG: dark grey columns; DTG: grey columns. Kinetics of 2-LTRc and 1-LTRc are shown in the middle

and right panels, respectively. WT: white diamonds; RAL: black squares; EVG: dark grey circles; DTG: grey diamonds. Each value corresponds to an average of five to six independent experiments and confidence intervals analysis are shown for a p value <0.05.

Figure S6: LM-PCR protocol. Dilutions of the standard curve as well as a sample of pLIN-HIV-ScaI digested by ScaI/AatII (1,000 copies) in triplicate were submitted to 8, 12 or 25 cycles for the first PCR. Then, amplified products were submitted to the second round of PCR. 8 cycles lead to a non-reproducible quantification of the sample due to weak amplification (left panel). 25 cycles lead to a loss in the linear range of the standard curve (right panel). 12 cycles are necessary for both reproducibility and accuracy of the quantification (middle panel). The same results were obtained using pLIN-HIV-NdeI digested by NdeI/AatII.

Table S1

A

Name	Oligonucleotide Sequence	Name of the linker
25t	5'-GCGGTGACCCGGGAGATCTGAATTC-3'	Linker 11b
11b	5'-GAATTCAGATC-3'	
25t	5'-GCGGTGACCCGGGAGATCTGAATTC-3'	Linker 11GTb
11GTb	5'-GTGAATTCAGATC-3'	
25t	5'-GCGGTGACCCGGGAGATCTGAATTC-3'	Linker 11TAb
11TAb	5'-TAGAATTCAGATC-3'	

B

Name	Oligonucleotide and Probe Sequence	Target	Denaturation	PCR cycles
MH 531	5'-TGTGTGCCCGTCTGTTGTGT	Total HIV-1 DNA	95°C, 8min	95°C 10s, 60°C 10s, 72°C 6s, for 50 cycles
MH 532	5'-GAGTCCTGCGTCGAGAGAGC			
MH FL*	5'-CCCTCAGACCCTTTTAGTCAGTGTGGAA[1]			
MH LC*	5'-TCTCTAGCAGTGGCGCCCGAACAG[2]			
HIV F	5'-GTGCCCGTCTGTTGTGTGACT	2-LTR circles	95°C, 8min	95°C 10s, 66°C 10s, 72°C 10s, for 15 cycles, then 55 cycles with the annealing temperature decreased by 0.5°C/cycle to 59°C
HIV R1	5'-ACTGGTACTAGCTTGTAGCACCATCCA			
HIV FL*	5'-CCACACACAAGGCTACTTCCCTGA[1]			
HIV LC*	5'-TGGCAGAACTACACACCAGGGC[2]			
L-M667	5'-ATGCCACGTAAGCGAAACTCTGGCTAACTAGGGAACCCACTG	Integrated HIV-1 DNA (first-round PCR)	95°C, 8min	95°C 10s, 60°C 10s, 72°C 170s, for 12 cycles
Alu1	5'-TCCCAGCTACTGGGGAGGCTGAGG			
Alu2	5'-GCCTCCCAAAGTGCTGGGATTACAG			
Lambda	5'-TATGCCACGTAAGCGAAACT	Integrated HIV-1 DNA (second-round PCR)	95°C, 8min	95°C 10s, 60°C 10s, 72°C 9s, for 50 cycles
AA55M	5'-GCTAGAGATTTTCCACACTGACTAA			
LTR FL*	5'-CACAACAGACGGGCACACACTACTTGA[1]			
LTR LC*	5'-CACTCAAGGCAAGCTTTATTGAGGC[2]			
32t	5'-GCGCGCGGCGGTGACCCGGGAGATCTGAATTC	Linear HIV-1 DNA (first-round PCR)	95°C, 8min	95°C 10s, 60°C 10s, 72°C 31s, for 12 cycles
MS1	5'-CTCGCCTCTTGCCGTGCGCG			
25t	5'-GCGGTGACCCGGGAGATCTGAATTC	Linear HIV-1 DNA (second-round PCR)	95°C, 8min	95°C 10s, 60°C 10s, 72°C 26s, for 50 cycles
MS2	5'-GAGTCCTGCGTCGAGAGATC			
MH FL*	5'-CCCTCAGACCCTTTTAGTCAGTGTGGAA[1]			
MH LC*	5'-TCTCTAGCAGTGGCGCCCGAACAG[2]			
1LTR-LA1	5'-GCGCTTCAGCAAGCCGAGTCCT	1-LTR circles	95°C, 8min	95°C 10s, 60°C 10s, 72°C 25s, for 50 cycles
1LTR-LA16	5'-GTCACACCTCAGGTACCTTTAAGACCAATGAC			
LTR FL*	5'-CACAACAGACGGGCACACACTACTTGA[1]			
LTR LC*	5'-CACTCAAGGCAAGCTTTATTGAGGC[2]			

1: Modified with fluorescein at the 3' end.
2: Modified with LC red 640 dye at the 5' end and phosphorylated at the 3' end.
Primers and probes were purchased from TIB MOLBIOL (Berlin, Germany).
* probe sequence.
Primers and probes used for for Total, 2-LTR circles and integrated viral DNA quantification were from reference 18
Primers used for Linear and 1-LTR circles quantifications were from reference 20 and 22, respectively.

Figure S1

A

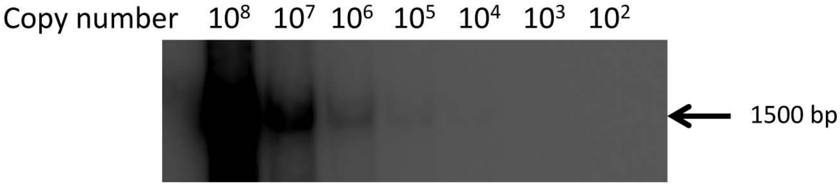
Copy number 10^8 10^7 10^6 10^5 10^4 10^3 10^2
1500 bp

B

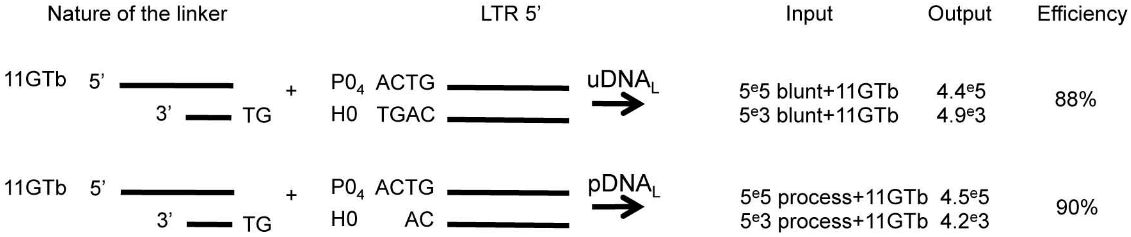
Nature of the linker
LTR 5'
Input
Output
Efficiency
11GTb 5'
3' TG
+
$P0_4$ ACTG
H0 TGAC
$uDNA_L$
5^e5 blunt+11GTb
5^e3 blunt+11GTb
4.4^e5
4.9^e3
88%
11GTb 5'
3' TG
+
$P0_4$ ACTG
H0 AC
$pDNA_L$
5^e5 process+11GTb
5^e3 process+11GTb
4.5^e5
4.2^e3
90%

Figure S2

elongation time: 18s

	input	output	%Amplification
1-LTRc	200000	90000	**45**
	20000	8233	**41**
	2000	187	**9**
	200	ND	**ND**
2-LTRc	200000	633	**0.3**
	20000	20	**0.1**
	2000	ND	**ND**
	200	ND	**ND**
Linear DNA	200000	24	**0.012**
	20000	ND	**ND**
	2000	ND	**ND**
	200	ND	**ND**

elongation time: 25s

	input	output	%Amplification
1-LTRc	200000	192500	**96.2**
	20000	21090	**105**
	2000	2002	**100**
	200	185	**92.5**
2-LTRc	200000	1383	**0.7**
	20000	160	**0.8**
	2000	38	**1.9**
	200	ND	**ND**
Linear DNA	200000	67	**0.03**
	20000	10	**0.05**
	2000	ND	**ND**
	200	ND	**ND**

elongation time: 32s

	input	output	%Amplification
1-LTRc	200000	190000	**95**
	20000	20234	**101**
	2000	1890	**94.5**
	200	201	**100.5**
2-LTRc	200000	27064	**13.5**
	20000	3580	**17.9**
	2000	128	**6.4**
	200	13	**6.7**
Linear DNA	200000	87047	**43.5**
	20000	5212	**26**
	2000	189	**9.5**
	200	48	**24**

Figure S3

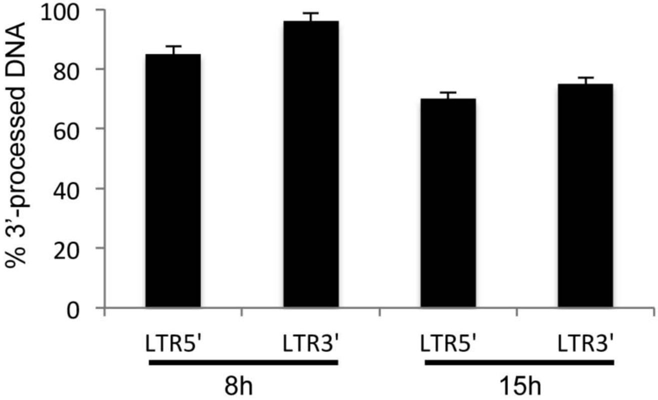

Figure S4

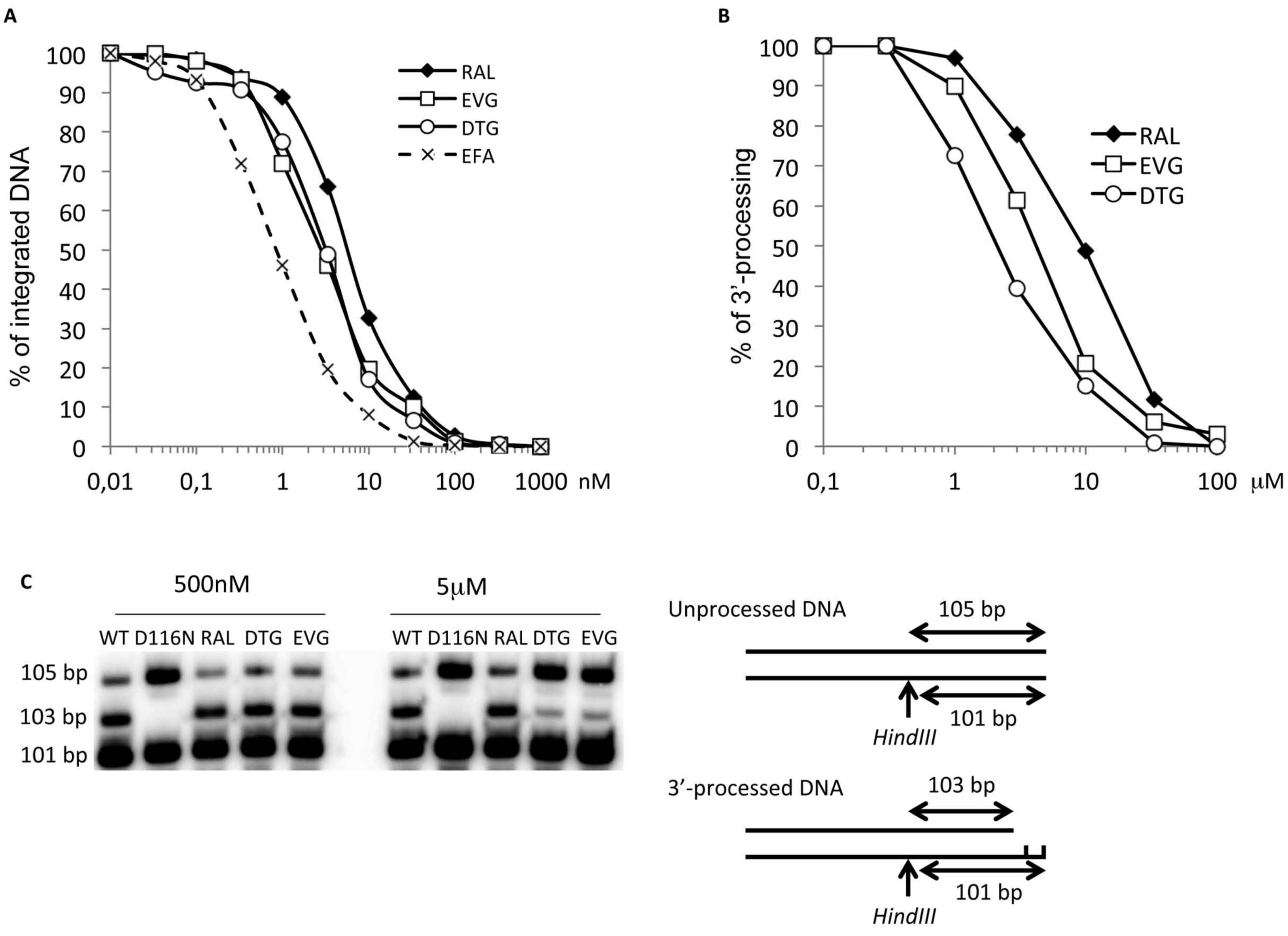
A
% of integrated DNA
100
90
80
70
60
50
40
30
20
10
0
0,01
0,1
1
10
100
1000
nM
RAL
EVG
DTG
EFA
B
% of 3'-processing
0,1
1
10
100
µM
RAL
EVG
DTG
C
500nM
5µM
WT D116N RAL DTG EVG
WT D116N RAL DTG EVG
105 bp
103 bp
101 bp
Unprocessed DNA
105 bp
101 bp
HindIII
3'-processed DNA
103 bp
101 bp
HindIII

Figure S5

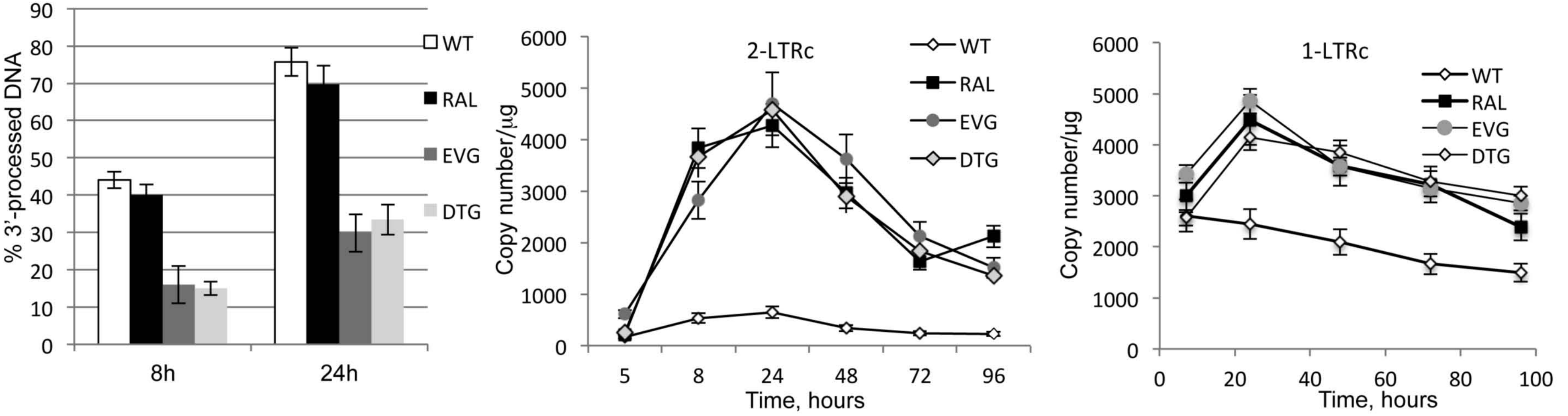

90
80
70
60
50
40
30
20
10
0
% 3'-processed DNA
8h
24h
WT
RAL
EVG
DTG
6000
5000
4000
3000
2000
1000
0
2-LTRc
Copy number/μg
5
8
24
48
72
96
Time, hours
WT
RAL
EVG
DTG
6000
5000
4000
3000
2000
1000
0
1-LTRc
Copy number/μg
0
20
40
60
80
100
Time, hours
WT
RAL
EVG
DTG

Figure S6

First PCR: 8 cycles

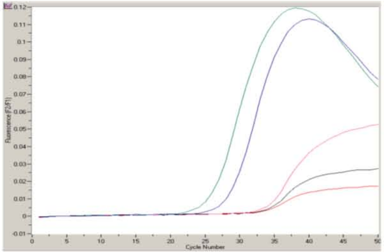

First PCR: 12 cycles

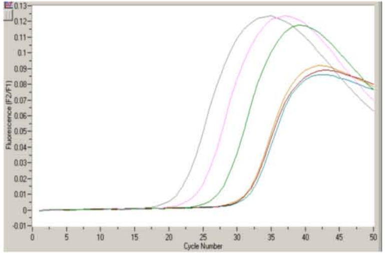

First PCR: 25 cycles

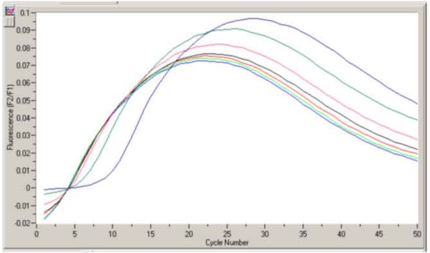

Supplement: Additional file 1 — The data sets supporting the results of this article are available: Table S1, Figures S1-S6. [file 1742-4690-10-87-S1.pdf]
